# Population genetic analysis reveals a geographically limited transition zone between two genetically distinct Atlantic salmon lineages in Norway

**DOI:** 10.1002/ece3.5258

**Published:** 2019-05-22

**Authors:** Vidar Wennevik, María Quintela, Øystein Skaala, Eric Verspoor, Sergey Prusov, Kevin A. Glover

**Affiliations:** ^1^ Institute of Marine Research Bergen Norway; ^2^ Rivers and Lochs Institute, Inverness College University of the Highlands and Islands Inverness UK; ^3^ The Knipovich Polar Research Institute of Marine Fisheries and Oceanography (PINRO) Murmansk Russia

**Keywords:** adaptation, isolation by distance, microsatellites, phylogenetics, Salmon

## Abstract

Atlantic salmon is characterized by a high degree of population genetic structure throughout its native range. However, while populations inhabiting rivers in Norway and Russia make up a significant proportion of salmon in the Atlantic, thus far, genetic studies in this region have only encompassed low to modest numbers of populations. Here, we provide the first “in‐depth” investigation of population genetic structuring in the species in this region. Analysis of 18 microsatellites on >9,000 fish from 115 rivers revealed highly significant population genetic structure throughout, following a hierarchical pattern. The highest and clearest level of division separated populations north and south of the Lofoten region in northern Norway. In this region, only a few populations displayed intermediate genetic profiles, strongly indicating a geographically limited transition zone. This was further supported by a dedicated cline analysis. Population genetic structure was also characterized by a pattern of isolation by distance. A decline in overall genetic diversity was observed from the south to the north, and two of the microsatellites showed a clear decrease in number of alleles across the observed transition zone. Together, these analyses support results from previous studies, that salmon in Norway originate from two main genetic lineages, one from the Barents–White Sea refugium that recolonized northern Norwegian and adjacent Russian rivers, and one from the eastern Atlantic that recolonized the rest of Norway. Furthermore, our results indicate that local conditions in the limited geographic transition zone between the two observed lineages, characterized by open coastline with no obvious barriers to gene flow, are strong enough to maintain the genetic differentiation between them.

## 1 INTRODUCTION

Sustainable management of biodiversity in exploited species requires among other things, an understanding of their structuring into distinct breeding populations, as well as the nature and extent of population connectivity and adaptive population differentiation. Elucidating connectivity among populations, and identifying the underlying mechanisms that shape observed patterns, represents an ongoing challenge. Given the ever‐increasing pressure on much of the world's biota and ecosystems, this is increasingly urgent. For the Atlantic salmon (*Salmo salar* L.), an iconic and economically important anadromous fish that has and continues to be subjected to a diverse array of anthropogenic challenges (Forseth et al., 2017; Glover et al., 2017; Parrish, Behnke, Gephard, McCormick, & Reeves, 1998; Taranger et al., 2015), it has never been more important to map populations, and quantify their evolutionary and contemporary relatedness and connectivity.

Atlantic salmon inhabit cold‐water rivers on both sides of the north Atlantic. In anadromous populations, the quintessential form, fertilized eggs are deposited in well‐oxygenated gravel areas, and after hatching, juveniles spend 1–5 + years in freshwater before migrating to the sea (Klemetsen et al., 2003; Metcalfe & Thorpe, 1990). After 1–3 + years of oceanic feeding, they mature and return to freshwater to reproduce, completing the life cycle. The species' anadromous life history involves long‐distance migrations from individual spawning rivers and tributaries to shared oceanic feeding areas where fish from multiple populations and regions meet (Bradbury et al., 2016; Gilbey et al., 2017; Olafsson et al., 2016; Sheehan, Legault, King, & Spidle, 2010), with all but a very small fraction of returning salmon, homing back to their natal rivers (Jonsson, Jonsson, & Hansen, 2003; Stabell, 1984). Accurate homing and fidelity to natal river provides the isolating mechanism through which genetically distinct populations have been able to establish in this species throughout its native range (Bourret et al., 2013; King, Kalinowski, Schill, Spidle, & Lubinski, 2001; Ståhl, 1987; Verspoor et al., 2005). In turn, this has also provided the basis for the evolution of genetic differences in life‐history traits among populations, some of which may be adaptive (Garcia de Leaniz et al., 2007; Taylor, 1991).

Atlantic salmon genetic population structure has been widely studied. Beyond the general conclusion that there is a high level of fine scale structuring, often to the tributary level (King, Eackles, & Letcher, 2005), in general, the genetic relationship among populations follows a hierarchical pattern. The largest genetic differences have been observed between populations inhabiting rivers on the east and west sides of the Atlantic (Gilbey, Knox, O'Sullivan, & Verspoor, 2005; Rougemont & Bernatchez, 2018; Taggart, Verspoor, Galvin, Moran, & Ferguson, 1995) and the smallest within rivers (King et al., 2005). At the extreme, salmon native to the American and European continents, show differences in chromosome number (Brenna‐Hansen et al., 2012; Lubieniecki et al., 2010). In general, within continents, population genetic structure is further divided into smaller geographical regions (Bourret et al., 2013; Cauwelier et al., 2018; Olafsson, Pampoulie, Hjorleifsdottir, Gudjonsson, & Hreggvidsson, 2014), and thereafter, among populations inhabiting rivers within regions (Perrier, Guyomard, Bagliniere, & Evanno, 2011; Tonteri, Veselov, Zubchenko, Lumme, & Primmer, 2009; Wennevik, Skaala, Titov, Studyonov, & Nævdal, 2004). Detailed accounts of structuring exist for some parts of the species range (King et al., 2007), including extensive recent accounts of microsatellite variation for southern Europe (Griffiths et al., 2010; Perrier et al., 2011), Iceland (Olafsson et al., 2014), Canada (Bradbury et al., 2015), and more recently, Scotland (Cauwelier et al., 2018). At the finest end of the scale, genetic differences have even been observed among tributaries within larger river systems (Dillane et al., 2007, 2008; Dionne, Caron, Dodson, & Bernatchez, 2009; Vaha, Erkinaro, Niemela, & Primmer, 2007).

Population genetic structure in Atlantic salmon is often, but not always, associated with isolation by distance (Dillane et al., 2007; Glover et al., 2012; Perrier et al., 2011; Primmer et al., 2006). To some extent, this will be because the level of contemporary straying among populations is a function of distance. However, other factors such as landscape features (Dillane et al., 2008), association with climate clines through local adaptation (Gilbey, Verspoor, & Summers, 1999; Jeffery et al., 2017; Verspoor, Fraser, & Youngson, 1991), and colonization history in connection with ice‐cap retreat patterns (Cauwelier et al., 2018; Olafsson et al., 2014; Rougemont & Bernatchez, 2018), play an important role in shaping population genetic structure in this species. Other factors may well be involved and all are likely to be of variable importance in defining levels of within and among river population differentiation.

Norway and Russia have approximately 400 and 110 rivers containing Atlantic salmon populations, respectively (http://www.nasco.int/RiversDatabase.aspx) and populations in this region represent a large proportion of the wild Atlantic salmon resources globally. Yet, despite the significance of this region for Atlantic salmon, a detailed picture of population genetic structure in Norway is lacking, with the literature on Norwegian rivers confined largely to scattered population samples within broader scale assessments (Bourret et al., 2013; Verspoor, 1997; Wennevik et al., 2004), although some Norway‐specific population genetic studies have also been published (Glover et al., 2013, 2012). Russian populations have been more extensively studied. Early studies using allozyme (Kazakov & Titov, 1991) and mitochondrial DNA markers (Makhrov, Verspoor, Artamonova, & O'Sullivan, 2005) described some of the major structuring of Atlantic salmon populations of the Russian north. However, several more recent studies, applying different classes of markers, have extended understanding of the population structure and the recolonization history of these northern populations since the last glaciation. Asplund et al. (2004), looked at mtDNA haplotype variation in 30 rivers from the eastern Barents Sea to the river Tana in Finnmark and suggested grouping the populations into three major clusters; one western group including the Barents Sea coast, one group including rivers from Kola Peninsula draining to the White Sea and an eastern group. In a study of Atlantic salmon populations from the Baltic, White and Barents Seas, Tonteri et al. (2005) concluded that it was most likely that the populations from the White and Barents Seas were colonized from multiple refugia, one possibly located in the eastern Barents Sea. In a follow‐up study with populations from the White and Barents Seas, Tonteri et al. (2009) found evidence of four distinct population clusters; Atlantic Ocean and western Barents Sea, Kola Peninsula, western White Sea and eastern Barents Sea. More recently, Ozerov et al. (2017) developed a high‐density genetic baseline for northern Atlantic salmon populations, and also briefly described population structure, identifying seven major population complexes, largely consistent with the results from the above‐mentioned studies.

The primary objective of the present study it is to provide the first detailed analysis of the population genetic structure of salmon stocks across the whole of Norway and western Russia. This analysis encompasses data for 9,165 salmon from 115 rivers analyzed for a panel of 18 microsatellite DNA markers. The secondary objective of this study is to place the data set in the public domain to facilitate comparative and integrated analyses of structuring patterns across the species’ range.

## 2 MATERIAL AND METHODS

### 2.1 Sampling

**Figure 1:**
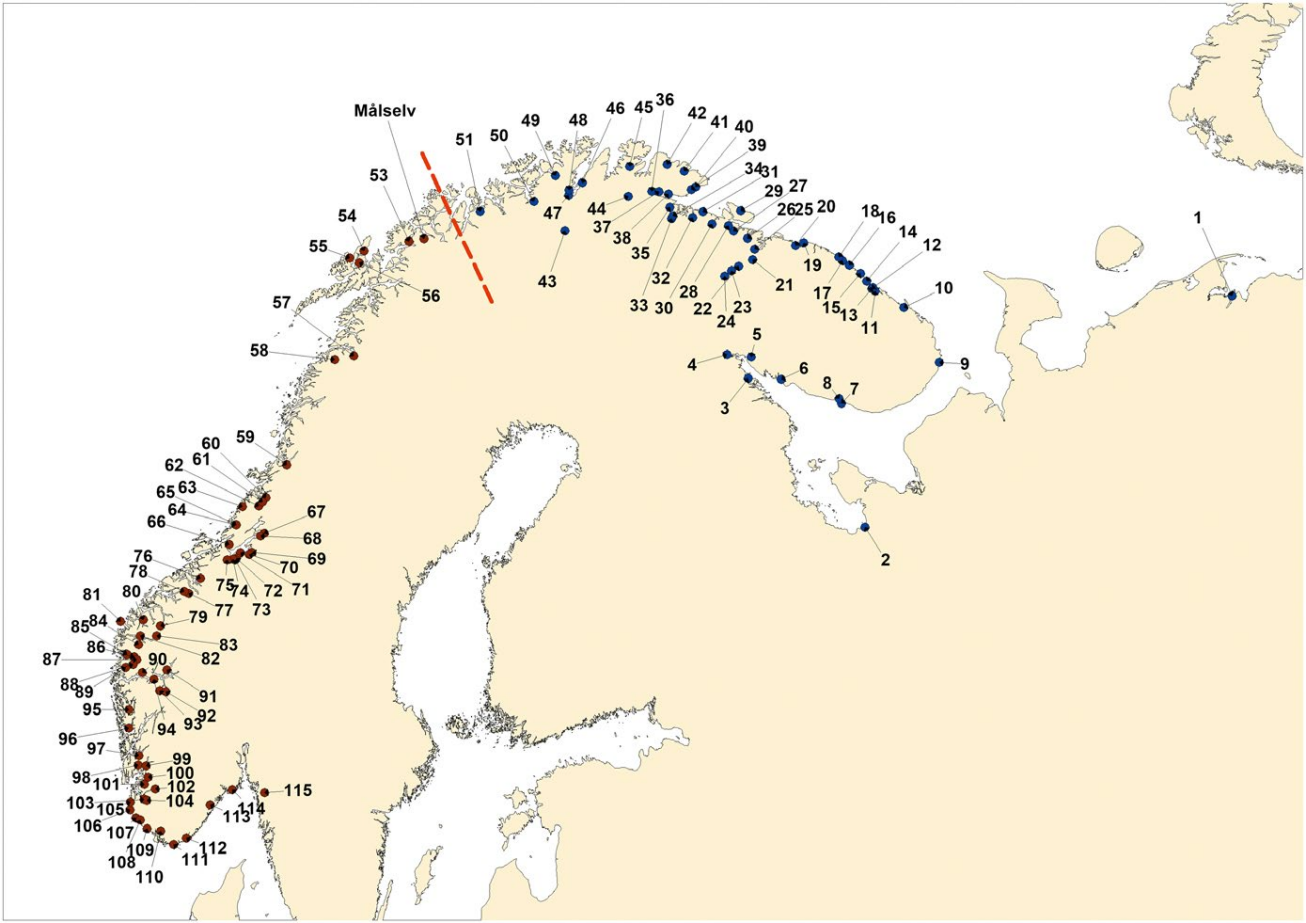
Map showing the location of the rivers sampled in Russia and Norway. Numbers refer to river names in Table A1. The major genetic division of the populations into two groups are indicated with a dashed line

In total, 9,165 individuals were sampled in 115 rivers from the Komi Republic in Russia to the Østfold region in southern Norway (Figure 1). This included samples of individuals from different stages of their life cycle (parr, fry, smolt, and adult), although most were of juveniles (fry & parr) collected by electrofishing at 2–4 locations within each river. In all cases, sampling encompassed individuals representing all juvenile year classes present at that particular sampling location. Fish were euthanized using an overdose of benzocaine, and fin clips were taken and transferred to tubes with 96% ethanol. Permits for collection of the samples were issued by County Governors in Norway, and by the Federal Agency for Fisheries in Russia. For simplicity, river samples are referred to as “population samples.”

### 2.2 Genotyping

DNA extraction was performed in 96‐well plates using the Qiagen DNeasyH96 Blood & Tissue Kit; each of which contained two or more negative controls. Eighteen loci were amplified in three multiplex reactions (full genotyping conditions available from authors upon request): SSsp3016 (GenBank no. AY372820), SSsp2210, SSspG7, SSsp2201, SSsp1605, SSsp2216 (Paterson, Piertney, Knox, Gilbey, & Verspoor, 2004), Ssa197, Ssa171, Ssa202 (O'Reilly, Hamilton, McConnell, & Wright, 1996), SsaD157, SsaD486, SsaD144 (King et al., 2005), Ssa289, Ssa14 (McConnell, O'Reilly, Hamilton, Wright, & Bentzen, 1995), SsaF43 (Sanchez et al., 1996), SsaOsl85 (Slettan, Olsaker, & Lie, 1995), MHC I (Grimholt, Drabløs, Jørgensen, Høyheim, & Stet, 2002), and MHC II (Stet et al., 2002). PCR products were analyzed on an ABI 3,730 Genetic Analyser and sized by a 500LIZ™ size standard. Automatically binned alleles were manually checked by two researchers prior to exporting data for statistical analysis. These markers have been extensively used in this laboratory for large‐scale pedigree reconstruction (Harvey, Glover, Taylor, Creer, & Carvalho, 2016; Solberg, Glover, Nilsen, & Skaala, 2013), forensic analysis (Glover, 2010; Glover, Skilbrei, & Skaala, 2008), ploidy validation (Glover et al., 2015; Jorgensen et al., 2018), and population analysis (Glover et al., 2012; Madhun et al., 2017). Thus, the data set is regarded as highly robust.

Data were screened using the software COLONY ver. 2.0.5.1 (Jones & Wang, 2010), which implements full‐pedigree likelihood methods to simultaneously infer sibship and parentage among individuals using multilocus genotype data, to purge the data set from full siblings that would lead to bias in allele frequency estimates as suggested by Allendorf and Phelps (1981), but see work by Waples and Anderson (2017). Analyses were run with no information on parental genotypes, assuming both male and female polygamy as well as possible inbreeding. The full‐likelihood model was chosen together with run length and precision set to medium. A total of 1,007 individuals were removed (Table A1).

### 2.3 Statistical analysis

Screening for outlier loci was performed using two methods. First, with the approach implemented in ARLEQUIN v.3.5.1.2 (Excoffier, Laval, & Schneider, 2005), which accounts for historical meta‐population structure with a hierarchical island model (*H*) (Excoffier, Hofer, & Foll, 2009) thus aiming to reduce the number of false positive *F*_ST_ outlier loci. The underlying assumptions are that the average migration rate between populations on different islands is lower than that between demes on the same island and that the heterozygosity between populations can be inferred using the heterozygosity within a population (Excoffier & Lischer, 2010). Significance of outliers was assessed by running 50,000 simulations, 100 demes, and 20 groups. Second, with the *F*dist approach (Beaumont & Nichols, 1996) implemented in LOSITAN (Antao, Lopes, Lopes, Beja‐Pereira, & Luikart, 2008) in which loci with an unusually high *F*_ST_ are considered to be putatively under directional selection. We simulated the neutral distribution of *F*_ST_ with 1,000,000 iterations at a significance level of 0.001 under a stepwise mutation model. This method also implements a multi‐test correction based on false discovery rates (FDR) to avoid high overestimation of the percentage of outliers (e.g., 1% of false positive with a threshold of 99%). Due to the impossibility of handling data sets exceeding 100 populations, LOSITAN was conducted separately for each of the regional divisions obtained from STRUCTURE.

Total number of alleles and allelic richness (*A*_r_) were calculated with MSA (Dieringer & Schlötterer, 2003), whereas observed (*H*_o_) and unbiased expected heterozygosity (*uH*_e_) were computed with GenAlEx (Peakall & Smouse, 2006). The genotype distribution of each locus per year class and its direction (heterozygote deficit or excess) was compared with the expected Hardy–Weinberg distribution using the program GENEPOP 7 (Rousset, 2008) as was the linkage disequilibrium. Both were examined using the following Markov chain parameters: 10,000 steps of dememorization, 1,000 batches and 10,000 iterations per batch. Significance was assessed after applying sequential Bonferroni correction (Holm, 1979). Effective population size (Ne) based on linkage disequilibrium was estimated using LDNe v1.31 (Waples & Do, 2008) using the random mating option and the *Pcrit* = 0.02 criterion for screening out rare alleles, and with 95% confidence intervals derived from a jack‐knife approach.

Allelic richness and heterozygosity were tested for latitudinal trends using the nonparametric Kendall measure of rank correlation (Kendall & Gibbons, 1976), which measures the similarity of the orderings of the data when ranked by north‐south gradient or by the value of the variable tested (Valz & Thompson, 1994), and implemented in the R Package “Kendall” (R Core Team, 2016). Besides, conservation limits (i.e., the number of spawning salmon needed for fully exploiting the rivers potential for production of juveniles) expressed as kg of female fish were tested for correlation with three different variables: *N*_e_, *H*_o_, and *A*_r_. River‐specific conservation limits information was only available for the Norwegian rivers.

Potential recent declines in effective population size were assessed using the software BOTTLENECK v1.2.02 (Piry, Luikart, & Cornuet, 1999) based on allele frequencies. As the data set was genotyped at <20 microsatellites, Wilcoxon's test and the graphical mode shift indicator were chosen (Piry et al., 1999). Likewise, loci were assumed to evolve under the two‐phase mutation model (Di Rienzo et al., 1994) with 5% of the mutations involving multiple steps with a variance of 12 (see Tonteri et al., 2009). Statistical significance of the Wilcoxon's test was assessed by 2,000 replications followed by the sequential Bonferroni correction for multiple significance tests.

Hierarchical population structure was explored using STRUCTURE (Pritchard, Stephens, & Donnelly, 2000) and traditional *F*_ST_ (Weir & Cockerham, 1984). STRUCTURE v. 2.3.4 was used to identify genetic groups under a model assuming admixture and correlated allele frequencies using population information to assist the analysis. STRUCTURE was analyzed following a hierarchical approach (Gilbey et al., 2017) using the program ParallelStructure (Besnier & Glover, 2013) that distributes jobs between parallel processors in order to significantly speed up the analysis time. Ten runs with a burn‐in period consisting of 250,000 replications and a run length of 750,000 MCMC iterations were performed for *K* = 1 to *K* = 20 clusters for the total data set. To determine the number of clusters in which samples could be divided into, the STRUCTURE output was analyzed by combining the visual inspection of the barplots with the ad hoc summary statistic Δ*K* of Evanno, Regnaut, and Goudet (2005), which is based on the rate of change of the “estimated likelihood” between successive *K* values and allows the determination of the uppermost hierarchical level of structure in the data. The data set was split into smaller units based upon this analysis until coherence in the clusters were lost, or until single rivers appeared as independent entities. Finally, runs for the selected *K*s were averaged with CLUMPP v.1.1.1 (Jakobsson & Rosenberg, 2007) using the LargeK Greedy algorithm and the G’ pairwise matrix similarity statistic and were graphically displayed using barplots. STRUCTURE allowed the partitioning of the data set into subsets of geographic regions that were analyzed in a hierarchical manner.

A Principal component analysis (PCA) was conducted using the program GenoDive, version 2.0b (Meirmans & Van Tienderen, 2004). The analysis was performed on populations (i.e., merged river samples) using a covariance matrix with 10,000 permutations. The results from the analysis were visualized as plots constructed in Microsoft Excel. The relationships among genetic distance and geographical distances were examined via a simple Mantel (1967) test between the matrices of pairwise *F*_ST_ and geographical distance. Mantel tests were conducted with PASSaGE (Rosenberg & Anderson, 2011), and significance was tested after 10,000 permutations. The program PGDSpider 2.1.1.3 (Lischer & Excoffier, 2012) was used to conduct the file conversion to the software used for the different analyses when required.

In order to further investigate the geographically limited transition zone identified by STRUCTURE (see results), we conducted a cline analysis to estimate the shape, center, and width of the cline generated by our molecular data (Gay, Crochet, Bell, & Lenormand, 2008). Geographic cline analysis over a 3,600 km transect starting in Unya in the Komi Republic in Russia to Enningdalselva in the Østfold region in the Norwegian–Swedish border were conducted with the R package HZAR (Derryberry, Derryberry, Maley, & Brumfield, 2014). The 15 models implemented in HZAR were fitted to the normalized loading of the first principal component analysis (PCA) axis based both on the panel of 18 microsatellites as well as on each locus independently to determine the position, width, and shape of clines over the total geographic distance. The reference cline was built using STRUCTURE Q‐score for the total data set and, in both cases, the best cline model was decided upon AIC scores. Clines were considered significantly displaced if the two log‐likelihood unit support limits of the cline center did not overlap with the STRUCTURE Q‐score (Qb = 1−Qs).

## 3 RESULTS

The raw genetic data for all of the individuals included in the present study are deposited in Appendix S1.

### 3.1 Genetic variation within populations

ARLEQUIN reported two outlier loci (Ssa289 and MHC2) in the full data set, whereas LOSITAN suggested that MHC2 was the only locus under directional selection in the two main clusters resulting after the first hierarchical division of the 115 samples. Thus, using a combined approach, MHC2 remained the only candidate for directional selection. The influence of this locus was tested by conducting STRUCTURE with and without it (Appendix S2). As inclusion/exclusion of this locus had no influence on the resulting genetic structure, MHC2 was retained in all the subsequent analyses.

Hardy–Weinberg deviations were reported in ~10% of the tests performed across populations for every locus, but they were reduced to 2.9% after sequential Bonferroni correction. Likewise, the percentage of deviations from LD decreased from 16.5% to 5% after correction. In both cases, the departures from expectations were distributed across populations and loci, therefore, no loci were dropped from the data set based on the results from these analyses.

Over the 18 microsatellites, a total of 413 alleles were observed, ranging from 5 to 7 alleles in SsaD486 and Ssa14, respectively, to 41 in SsaD144 and SsaD157. The total number of alleles per population ranged from 78 to 259, with a mean of 217 (Table A1). The Kovda(3) river showed an extremely low number of alleles: 78 in 26 individuals whereas, for example, 190 alleles were reported from 24 individuals sampled in the river Soknedalselva(109). The average number of alleles per locus within a population ranged from 4.3 in Kovda(3) to 14.4 in Eidselva(82), whereas allelic richness ranged from 4.3 in Kovda(3) to 10.76 in Otra(112).

**Figure 2:**
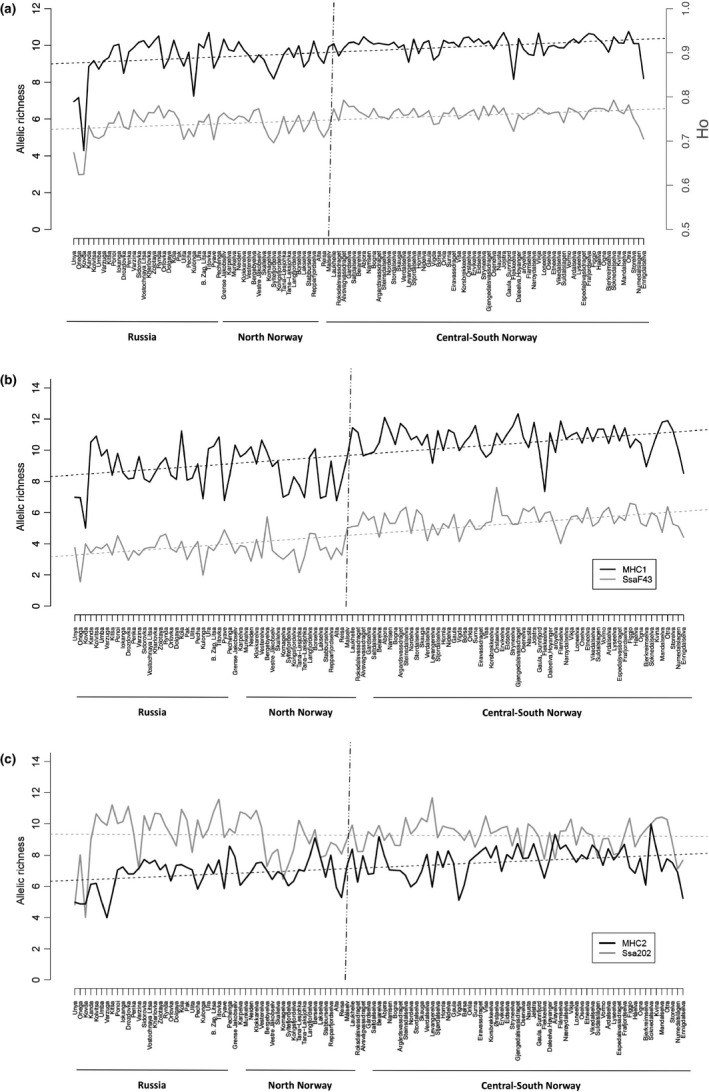
Patterns of genetic diversity assessed along the coastline from Russia to southern Norway. Genetic diversity was measured as: (a) Ho and overall allelic richness (Ar) per population; and (b) Ar for two out of the eleven loci (Sp1605, SsaF43, MHC1, SsaD486, SSspG7, Ssa14, Ssa289, MHC2, SsaD157, SSsp2210, Ssa197) showing significant trends. (c) Ar for MHC2 and SS202 (the second one showing no significant trend, for the sake of the comparison). Both the overall Ar for the 18 loci (*τ* = −0.281, *p* = 9.37*e*−06) and Ho (*τ* = −0.255, *p* < 0.001) experienced a significant decline from south to north. The vertical dashed line shows the first level of STRUCTURE division of the data set

The level of genetic variation showed a significantly increasing latitudinal N‐S trend following the coastline from Russia to southern Norway when measured either as: average number of alleles per locus within population (*τ *= −0.177, *p* = 0.005), *Ho* (*τ* = −0.255, *p* < 0.0001), *uHe* (*τ* = −0.36, *p* < 0.0001) or overall allelic richness (*τ* = −0.281, *p* < 0.0001) (Figure 2). The same pattern of *A*_r_ was statistically significant for 10 out of the 18 loci screened (i.e., SsaF43, MHC1, SsaD486, SSspG7, Ssa14, Ssa289, MHC2, SsaD157, SSsp2210, and Ssa197) whereas for locus Sp1605, the trend was reverse (*τ* = 0.3, *p* < 0.0001).

In Norway, the conservation limits expressed as kg of female fish per river were significantly correlated with Ne (*r* ^2 ^= 0.2085, *p* < 0.001), but not with Ho (*r* ^2 ^= −0.01, *p* = 0.68) nor with Ar (*r* ^2 ^= 0.004, *p* = 0.25). After performing Bonferroni correction for multiple comparisons, the Wilcoxon test did not reveal any population displaying evidence of having experienced recent bottlenecks. Likewise, no mode shift in allele frequencies was detected in any of them, all showing L‐shaped allele frequency distributions; that is, the number of alleles in the low‐frequency classes (<0.1) exceeded the number of alleles in the higher frequency ones.

### 3.2 Among‐population genetic structure

**Figure 3:**
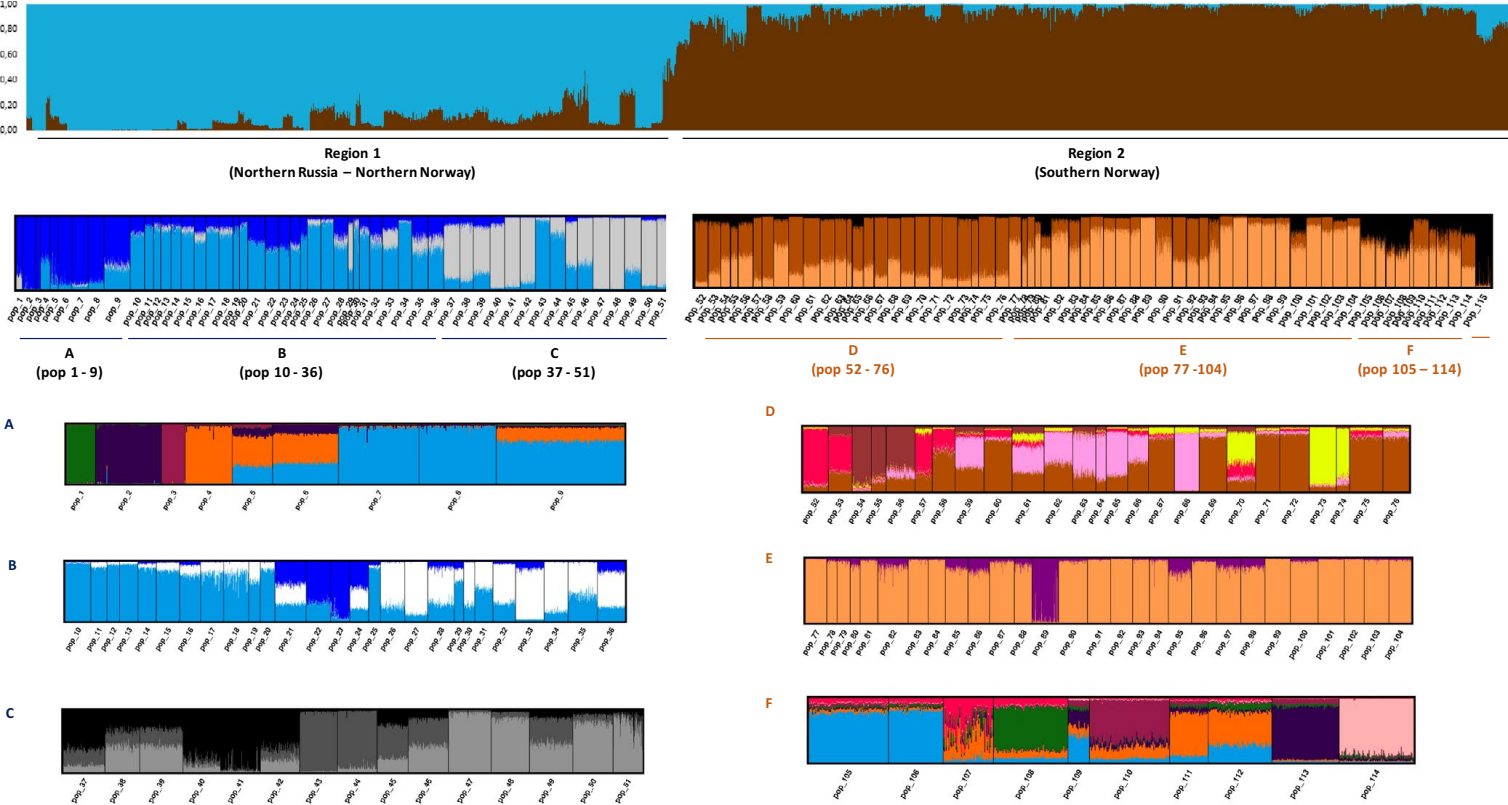
Hierarchical Bayesian clustering of the 115 populations using locprior information in structure

The first hierarchical level of division detected by Δ*K* test of STRUCTURE results showed two clusters (Δ*K* = 176.5) that divided the data set in a northernmost group ranging from the rivers Unya(1) to Reisa(51) (i.e., 51 sampled rivers), and a southern cluster from the rivers Laukhelle(53) to Enningdalselva(115) (63 rivers) (Figure 1). The ancestry of the population in the river Målselva(52) was almost evenly split between both clusters. At the second hierarchical level of division, further structure was revealed among populations (Figure 3). In the northern group, the eastern populations from Unya(1) to Kitsa(8) formed a distinct cluster in the plots from the structure analysis, different from the populations draining to the Barents Sea coast on the northern side of the Kola Penisula. The river Ponoi(9) appears as a transitional river. This genetic division also corresponds to a change in life history as the eastern populations and the White Sea populations are mainly “autumn‐run” salmon, which ascend the river the more than a year before spawning, while the Barents Sea rivers are dominated by “summer‐run” salmon spawning in the same year they return to the river. On the northern coast of the Kola Peninsula, there seems to be a genetic shift between the rivers east (10–20) and west 26–36 of the Kola Bay (Figure 3b). The rivers draining into the freshwater Tuloma lake (21–24) form a distinct cluster. Another genetic shift can be observed between rivers Bergebyelva(36) and Vestre Jakobselv(37) in the inner part of the Varanger Fjord. The two Tana tributaries Iesjohka(43) and Laksjohka(44) also appear different and distinct from neighboring rivers (Figure 3c). In the southern group, the rivers from Laukhelle(53) to Surna(76) appear fairly similar at *K* = 3 in the structure plot; however, the island rivers Roksdalsvassdraget(54), Alvsvågvassdraget(55), and Gårdselva(56) appear different from the rivers on the mainland. This was revealed more clearly at structure runs at higher values of *K* (Figure 3d). In the Trondheimsfjord, similarities can be seen between the larger salmon populations (67, 69, 71, 72 and 75) while the smaller rivers appear different (Figure 3d). Further south, a genetic division was observed between the rivers from Eiravassdraget(77) to Frafjordselva(104) and the more southern/eastern rivers. The rivers Numedalslågen (114) and Enningsdalselva(115) were distinct and different from other rivers in this southernmost region, while the rivers Figgjo(105) and Håelva(106), both draining directly into the ocean, show similarities.

**Figure 4:**
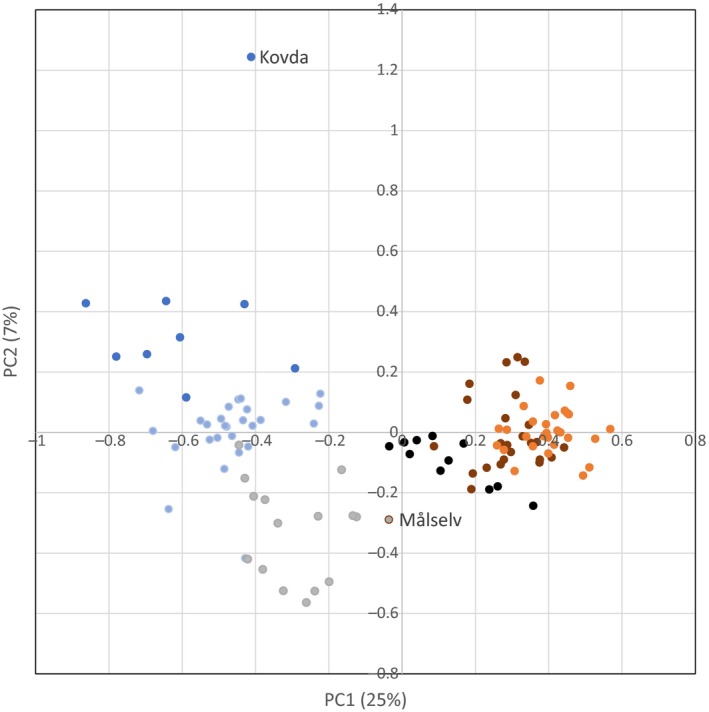
PCA plot of all 115 Atlantic salmon populations included in the analysis. The color coding corresponds to the major clusters detected in the STRUCTURE analysis, where dark blue is region 1.1 (Unya‐Ponoi), light blue is region 1.2 (Iokanga‐Vesterelva), gray is region 1.3 (Bergebyelva‐Reisa), brown is region 2.1 (Målselv‐Surna), orange is region 2.2 (Eira‐Frafjordelvaelva), and black is region 2.3–2.4 (Figgjo‐Enningdalselva)

Results of the PCA analysis (Figure 4) were consistent with the geographical defined genetic clusters resolved by structure (Figure 3). The first PC described 26% of the variation along a mainly north‐south gradient and separated the two main clusters clearly, with Målselva(52) appearing as a transitional population between the two main groups. The second PC described 7% of the variation and separated the three main clusters within the northern group, with the Kovda(3) population appearing as an outlier. The three main clusters within the southern group were less clearly separated by this analysis.

All global single‐locus estimates for *F*_ST_ were statistically different from zero (*p* < 0.0001), ranging between 0.012 (SsaD486) and 0.079 (Ssa289), with the global estimate over the 18 loci being 0.037 (*p* < 0.0001). The highest pairwise *F*_ST_ (0.202) was identified between the two Russian rivers Unya(1) and Kovda(3) (see Appendix S2 for complete matrix), located 1,236 km apart. The lowest pairwise *F*_ST_ values (<0.001) were recorded between five pairs of rivers within a range of 19 to 430 km of distance from each other. Almost all the pairwise comparisons except for 14 (0.2%) were significantly different from zero (*p* < 0.05). The nonsignificant values ranged from 0.0007 to 0.0041 in a range of geographic distances of 19–534 km.

**Figure 5:**
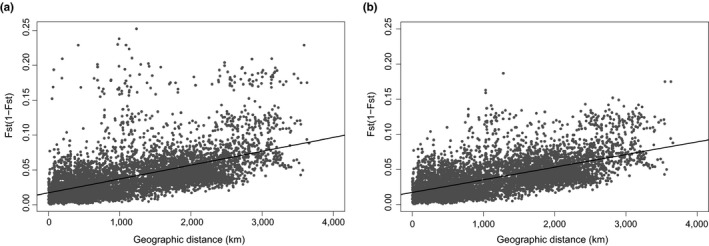
Relationship between geographic distance (km) and genetic distance measured as *F*_ST_/(1−*F*_ST_). Mantel test revealed a significant pattern of Isolation by Distance when using the full data set (a: *r* ^2 ^= 0.295, *p* < 2.2*e*−16) that became stronger when removing Kovda from the analyses (b: *r* = 0.618, *p* < 0.0001)

A Mantel test revealed a positive association between genetic distance measured as *F*_ST_ and geographic distance, demonstrating an overall pattern of genetic isolation by distance (IBD) among the 115 populations (*r* = 0.562, *p* < 0.0001, Figure 5a). The upper cluster of points in this graph corresponds mainly to the pairwise comparisons between samples from the Russian river Kovda(3) and other samples (*F*_ST_ values from 0.1321 to 0.20). The removal of the aberrant Kodva(3) sample increased the strength of the IBD pattern (r = 0.618, *p* < 0.0001, Figure 5b).

### 3.3 Investigation of the transition zone by cline analysis

**Figure 6:**
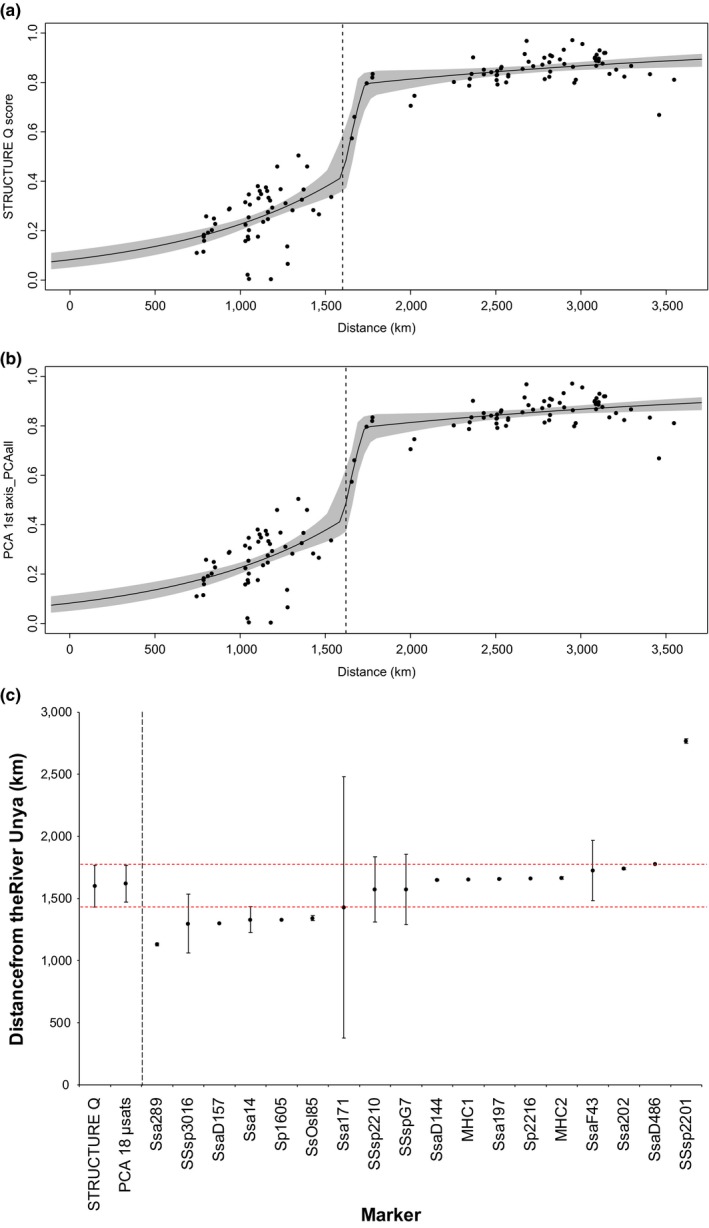
Geographical cline analysis for Atlantic salmon across a 3,600 km transect ranging from the Komi Republic in Russia to the Østfold region in the Norwegian‐Swedish border. Shape of the cline for the (a) STRUCTURE Q‐score and (b) the normalized loading on the first PCA axis based on the panel of 18 microsatellites with the narrow 95% credible cline region shaded in gray, and center of the cline depicted by the vertical dashed line. Furthermore, (c) position of the clines (center and width) for the STRUCTURE Q‐score, the normalized loading on the first PCA axis based on the panel of 18 microsatellites and on each locus separately. Red dashed lines depict the width of the STRUCTURE reference cline

The PCA cline based on the total 18 microsatellites fitted a fixB model, with the center situated at 1,621 km from the Unya(1) and with a width of 296 km (Figure 6, Table S1—Appendix S2). Both the center and the width of this cline were geographically located between the rivers Reisa(51) and Målselva(52), in very close agreement with the results from STRUCTURE (Figure 3). The PCA cline overlapped with the STRUCTURE Q‐score cline, which also met a fixB model, with the center located at 1,600.6 km (also between rivers Reisa(51) and Målselva(52)) and 336.4 km of width. The clines generated by the microsatellite loci SSsp2210, SSspG7, SsaD144, MHC1, Ssa197, Sp2216, MHC2, SsaF43, and Ssa202 presented their centers within the width of the reference cline based on the STRUCTURE Q‐score. Loci SsaD486 and SSsp2201 showed clines centered further south, unlike loci Ssa289, SSsp3016, SsaD157, Ssa14, Sp1605, SsOsl85, and Ssa171, which were centered between the rivers Kovda(3) and Tana‐Iesjohka(43). The graphical representation of the clines computed for each marker separately is shown in Appendix S3—Figure S1.

## 4 DISCUSSION

This study, based on the analysis of >9,000 individuals sampled in 115 rivers, represents the first extensive investigation of genetic structure within and among Norwegian and northwest Russian Atlantic salmon populations. Our most important results are summarized as follows. We observed (a) highly significant population genetic structuring in all regions, following a hierarchical geographic pattern, (b) a clear genetic division in the north of Norway with a geographically limited transition zone (Figures 3 and 6), (c) population genetic structure further influenced by a pattern of isolation by distance across the entire study area, and (d) a decline in genetic variation within populations from the south to the north, with two of the microsatellites showing a clear decrease in number of alleles across the identified transition zone.

Based on the main observations detailed above, we conclude that Atlantic salmon in Norway originate mainly from two genetic lineages, one from the Barents–White Sea refugium that recolonized northern Norwegian and adjacent Russian rivers, and one from the eastern Atlantic that recolonized the rest of Norway. We also conclude that local conditions in the geographically limited transition zone between these two lineages in northern Norway, characterized by a relatively open coastline with no obvious barriers to straying nor gene flow, are strong enough to maintain its character since its post‐last glacial maximum establishment. Whether or not selection, restricted straying and gene flow, or other mechanisms are responsible for its maintenance, remains to be elucidated.

### 4.1 Phylogeographic patterns in northern Norway and northwest Russia

The distinctiveness of salmon in northern Norway and northwest Russia, as compared to other European regions, was first noted in a study of 15 rivers across the species’ range using allozyme markers (Bourke, Coughlan, Jansson, Galvin, & Cross, 1997). This has also been observed in subsequent studies applying different classes of molecular markers (Bourret et al., 2013; Gilbey et al., 2017; Ozerov et al., 2017; Rougemont & Bernatchez, 2018; Skaala et al., 1998; Tonteri et al., 2009). However, the number of rivers included in some of these studies was limited. The first study to report a more precise geographic location of the clear genetic break in Norway, potentially reflecting the recolonization ranges from different lineages, was a study of microsatellite genetic variation in 21 Norwegian populations (Glover et al., 2012). These authors identified a genetic division in the geographic region between Målselva and Roksdalsvassdraget (populations 52 and 54 in the present study) which is consistent with the division revealed from the analysis here (Figure 3). Subsequent studies with SNPs, primarily aimed at investigating introgression of domesticated Atlantic salmon escapees in Norwegian populations, have also detected a distinct genetic change in this region (Glover et al., 2013; Karlsson, Diserud, Fiske, & Hindar, 2016).

The existence of a clear genetic divide in northern Norway is most likely to reflect the postglacial colonization history of this region, and the influence of mechanisms maintaining this divide over time. As mentioned above and in the introduction, several studies have suggested that the northern areas of Russia and Norway were colonized by different lineages, originating from different refugia. The eastern part of the Barents Sea was not entirely covered by ice during the last glacial maximum (Hughes, Gyllencreutz, Lohne, Mangerud, & Svendsen, 2016), and this area has been suggested as the location of a refugium from which the northeastern part of the distribution range of Atlantic salmon was colonized (Asplund et al., 2004; Kazakov & Titov, 1991; Nilsson et al., 2001; Rougemont & Bernatchez, 2018; Tonteri et al., 2005, 2009). Asplund et al. (2004) suggested that populations east of the genetic divide, observed in the eastern part of the Kola peninsula (this divide also shown by Tonteri et al., 2009 and present in our data set), primarily originated from this eastern refugium, while populations on the northern side of the peninsula and westwards into northern Norway mainly originated from other Atlantic lineages. Our data are consistent with this.

Several studies have demonstrated the presence of North American alleles/haplotypes in populations along the Barents Sea coast (Asplund et al., 2004; Bourke et al., 1997; Makhrov et al., 2005; Nilsson et al., 2001; Rougemont & Bernatchez, 2018) suggesting a contribution from both eastern and western Atlantic lineages. Based on a joint analysis of both Esterase‐D* and mtDNA, Mahkrov, and colleagues (Makhrov et al., 2005) first proposed that the genetic affinities of the region's salmon populations to those in North America arose from the unique postglacial recolonization of the area by salmon from both Europe and North America. In combination with other observations (Asplund et al., 2004; Tonteri et al., 2009), a geographical cline in western Atlantic genetic types suggests that the western Barents Sea/northern Kola Peninsula rivers may represent a further zone of secondary contact between eastern and western Atlantic lineages colonizing this area, in addition to the transition zone between this area and southern Norway. This possibility needs to be explored by a more in‐depth genetic analysis as recently reported for the zone of secondary contact between European and North American salmon in eastern Canada (Lehnert et al., 2018).

### 4.2 The geographically sharp transition zone between the eastern Atlantic and Barents–White Sea lineages

The continued existence of a geographically limited transition zone in northern Norway between two highly divergent regional salmon lineages raises both evolutionary and ecological questions. From an ecological perspective, do the evolved differences in the two regional groups encompass significant differences in their biologies, and what mechanisms maintain this geographically sharp divide? We suggest that there are potentially two mechanisms that interlink: (a) restricted straying and/or gene flow, (b) divergent selective forces.

There are still many unknowns regarding straying rates among salmon populations, though they clearly vary in time and space (Jonsson et al., 2003; Pedersen, Rasmussen, Nielsen, Karlsson, & Nyberg, 2007; Skilbrei & Holm, 1998; Stabell, 1984), and while some knowledge has been gained in recent years on their marine migration behavior (Chittenden, Adlandsvik, Pedersen, Righton, & Rikardsen, 2013; Gilbey et al., 2017; Gudjonsson, Einarsson, Jonsson, & Gudbrandsson, 2015; Strøm, Thorstad, Hedger, & Rikardsen, 2018), a large number of questions remain with respect to their oceanic migration routes and offshore feeding areas. Nevertheless, a lack of synchrony in marine growth of salmon populations from northern versus western Norway suggest that salmon originating from these two regions may utilize different oceanic feeding areas (Jensen et al., 2011). If this is the case, then fish retuning to the coastline in the region just north and south of the geographically limited transition zone identified here may come from different directions/oceanic areas, and act to reduce straying between the two regions. In turn, this could limit gene flow. However, a study of straying from two populations north of this transition zone found that fish strayed into rivers south of it (Ulvan et al., 2018), suggesting that the occurrence of some genetic mixing cannot be ruled out.

We observed a decrease in several estimators of genetic diversity with an increase in latitude (Figure 2a) and a clear “shift” in allelic variation at two of the genetic markers in the transition zone where the aforementioned lineages meet (Figure 2b). In Canadian Atlantic salmon populations, a gradient in genetic diversity and allelic variation at the MHC2 locus has been reported (Dionne, Miller, Dodson, Caron, & Bernatchez, 2007), and a relationship between allele frequencies and latitude was observed for immune‐related genes among European Atlantic salmon populations (Tonteri, Vasemägi, Lumme, & Primmer, 2010). Furthermore, allelic gradients with latitude and temperature have also been observed in respect of allelic variation at the MEP‐2* locus on both sides of the Atlantic both within and among rivers (Verspoor & Jordan, 1989). A recent study using whole genome resequencing identified functional genetic differences between salmon populations from the north and the rest of Norway (Kjaerner‐Semb et al., 2016), with evidence of islands of divergence on chromosomes 5, 10, 11, 13–15, 21, 24, and 25, possibly resulting from divergent selection regimes. This divergence included 59 known genes, 15 of which displayed one or more differentiated missense mutations. The strongest of these islands of divergence, located on chromosomes 25 and 5, respectively, contained genes involved in anti‐viral and pathogen control. It is not possible to conclude the functional significance of the clear general decrease in genetic diversity as revealed in the present study, or specifically for two of the markers across the observed transition zone. While clearly further work is needed, what evidence there is points to the possibility of functional genetic differences between populations in these two regions, possibly arising from a combination of differences relating to phylogenetic background and lineage recolonization, and divergent selection regimes. If as suggested, divergent selection regimes between these areas exist, even if some interbreeding does occur due to straying across the transition zone, reduced survival of the “nonlocal” type, as observed across watercourses in Ireland (McGinnity et al., 2004), may strongly constrain effective gene flow and help maintain geographically restricted transition zone.

### 4.3 Patterns of population genetic connectivity

A hierarchical pattern in genetic structure as revealed here, that is, within and among‐regional levels of differentiation (Figures 3 and 4), also characterized by an overall pattern of isolation by distance (IBD) (Figure 5), is a typical feature of Atlantic salmon populations (Glover et al., 2012; Tonteri et al., 2009; Vaha, Erkinaro, Falkegard, Orell, & Niemela, 2017). In addition to the highest level regional differentiation in northern Norway, a further less marked splitting of the Barents–White Sea and eastern Atlantic lineages and several other genetic sub‐groups was resolved (Figure 2). A second order division in population structure was reported in the Kola Peninsula of Russia between samples from the Ponoi and Iokanga (populations 9 and 10 in Figure 3). This corresponds to the division reported in earlier studies (Ozerov et al., 2017; Saisa et al., 2005; Tonteri et al., 2009) and to changes in the life‐history pattern of populations (Berg, 1948). Other genetic divisions were also revealed (Figure 3), illustrating the existence of both long‐distance and regional levels of genetic structure.

Population size differences (as evaluated from catch statistics or conservation limits), and potentially life history or other adaptive characteristics, appear linked with some of the patterns of genetic structure observed here. For example, in the relatively isolated Trondheimsfjord in mid‐Norway, the ten rivers sampled show a clear pattern of genetic divergence between the rivers with demographically small populations (populations 66, 68, 70, 73, 74) and those with demographically large or very large populations (populations 67, 69, 71, 72, 75) (Figure 3). This effect is also apparent in respect of the rivers Gaula and Orkla, which are genetically very similar to each other (populations 72 and 75), yet very distinct from the two small populations located between them, Vigda and Børsa, that are also similar to each other (populations 73 and 74) (Figure 3). It is thus striking that the two very large rivers have not dominated or overridden the genetic characteristics of these two much smaller populations, something observed in studies in other regions (Verspoor, 2005; Verspoor, Knox, & Marshall, 2016), once again suggesting a role for adaptive divergence even on a local scale.

Landscape features are known to influence population genetic structure in Atlantic salmon (Dillane et al., 2008; Ozerov, Veselov, Lumme, & Primmer, 2012). Although beyond the scope of this study, obvious landscape features also appeared to be linked with some of the population genetic structure revealed here. For example, on the coastline of Jæren on southwestern Norway, a genetic divide was revealed among populations in the Boknafjord region (populations 100–104) versus the immediately neighboring open coastline stretch of Jæren (populations 105–109) (Figure 3). Also, the rivers located in the relatively isolated Trondheimsfjord area showed differentiation to rivers on the outside of this fjord area, which overlays the significant observations within the fjord as discussed above (Figure 3). Thus, there is considerable evidence that the evolutionary relationships among populations and their genetic differentiation is driven by more than just historical and contemporary gene flow conditioned by IBD.

## CONFLICT OF INTERESTS

The authors declare that they have no competing interests.

## AUTHORS' CONTRIBUTIONS

VW, ØS, SP, and KAG conceived the study. VW and SP conducted/organized sampling of rivers. MQ and VW conducted statistical analyses and drafted part of the text. VW and SP provided background material to the study. EV assisted in data interpretation and finalizing the manuscript. VW and KAG led the process of data interpretation and development of the final version of the manuscript, with scientific contributions from all other authors. All authors read and approved the final manuscript.

## ETHICAL APPROVAL

Samples (fin clippings) were obtained from juvenile fish caught by electrofishing, or in some cases, scale samples collected in rod fisheries. Juvenile fish captured by electrofishing were euthanized before samples were collected. The permits required to obtain samples were issued by the Federal Agency for Fisheries (Russian Federation), and different County Governors of Norway.

## Data Availability

Appendices S1–S3 with data and supporting information, including the entire raw data set of individual genetic profiles for >9,000 salmon can be downloaded from the Norwegian Marine Data Centre (NMDC): https://doi.org/10.21335/NMDC-290855015.

## APPENDIX A

**Table A1:** Summary statistics per sample: Geographic coordinates per river; type of sample; total number of individuals, and number of individuals once full siblings have been removed; total number of alleles; allelic richness (AR, based on a sample of minimum 24 diploid individuals), number of private alleles; observed heterozygosity, Ho (mean ± *SE*); unbiased expected heterozygosity, uHe (mean ± *SE*); inbreeding coefficient, *F*_IS_ (mean ± *SE*); number (and percentage) of deviations from Hardy–Weinberg equilibrium (HWE) at *α* = 0.05; number (and percentage) of deviations from Linkage Disequilibrium (LD) at *α* = 0.05 and effective population size (Ne) with 95% confidence interval obtained by jack‐knife method (in brackets) calculated using the random mating option and the Pcrit = 0.02 criterion for screening out rare alleles

No	River	Latitude	Longitude	Sample type	No samples	No samples after fullsib removal	No total alleles	Ar	No private alleles	Ho	uHe	*F*_IS_	No (%) dev LD at *p* < 0.05	No (%) dev HWE at *p* < 0.05	Ne (CI)
1	Unya	68.2122	54.2189	Parr/fry	48	32	131	6.95	0	0.67 ± 0.06	0.66 ± 0.06	−0.040 ± 0.027	11 (7.2)	1 (5.6)	48.4 (35.8, 71.3)
2	Onega	63.9167	38.0000	Smolt	88	72	157	7.16	0	0.62 ± 0.06	0.65 ± 0.06	0.031 ± 0.017	19 (12.4)	3 (16.7)	84.4 (66.8, 111.1)
3	Kovda	66.6833	32.8500	Parr/fry	47	26	78	4.29	0	0.63 ± 0.04	0.60 ± 0.04	−0.047 ± 0.027	19 (12.4)	2 (11.1)	23.3 (16.2, 36.4)
4	Kanda	67.1292	31.9053	Parr/fry	55	52	186	8.85	0	0.74 ± 0.06	0.72 ± 0.05	−0.027 ± 0.015	10 (6.5)	2 (11.1)	207.7 (139.7, 384.6)
5	Kolvitsa	67.0833	32.9833	Parr/fry	45	44	186	9.17	0	0.71 ± 0.06	0.73 ± 0.06	0.011 ± 0.012	11 (7.2)	0	236.8 (151.4, 508.6)
6	Umba	66.6667	34.3000	Parr/fry	89	72	192	8.71	0	0.71 ± 0.06	0.71 ± 0.06	0.001 ± 0.015	8 (5.2)	3 (16.7)	256.9 (174.1, 465.4)
7	Varzuga	66.2167	36.9667	Parr/fry	91	88	213	9.16	2	0.71 ± 0.06	0.72 ± 0.06	−0.006 ± 0.014	10 (6.5)	2 (11.1)	1,172.7 (488.1, Infinite)
8	Kitsa	66.3020	36.8620	Parr/fry	96	85	211	9.34	1	0.74 ± 0.06	0.74 ± 0.06	−0.014 ± 0.012	7 (4.6)	0	298.8 (221.9, 446.7)
9	Ponoi	66.9833	41.2833	Parr/fry	141	140	251	9.98	1	0.74 ± 0.06	0.75 ± 0.06	0.012 ± 0.010	10 (6.5)	1 (5.6)	1,336.8 (715, 7,956.6)
10	Iokanga	68.0000	39.7167	Parr/fry	78	77	230	10.06	0	0.77 ± 0.06	0.75 ± 0.06	−0.028 ± 0.016	12 (7.8)	1 (5.6)	1,244.1 (562, Infinite)
11	Drozdovka	68.3000	38.4500	Parr/fry	63	49	174	8.48	0	0.73 ± 0.06	0.73 ± 0.06	−0.008 ± 0.013	7 (4.6)	0	96.4 (78.3, 123.4)
12	Penka	68.3500	38.3000	Parr/fry	42	38	195	9.64	0	0.73 ± 0.06	0.74 ± 0.06	−0.005 ± 0.018	5 (3.3)	1 (5.6)	393.2 (180.4, Infinite)
13	Varzina	68.3667	38.3500	Parr/fry	61	55	214	9.88	0	0.77 ± 0.06	0.76 ± 0.06	−0.029 ± 0.019	4 (2.6)	0	1,198.9 (448.7, Infinite)
14	Sidorovka	68.4833	38.0833	Parr/fry	74	55	217	10.17	0	0.75 ± 0.05	0.78 ± 0.05	0.005 ± 0.012	46 (30.1)	2 (11.1)	1,227.1 (406.2, Infinite)
15	Vostochnaya Litsa	68.6333	37.8000	Parr/fry	87	70	233	10.25	0	0.74 ± 0.06	0.76 ± 0.06	0.009 ± 0.018	9 (5.9)	2 (11.1)	279.8 (207.4, 420.8)
16	Kharlovka	68.7833	37.3167	Parr/fry	76	63	221	9.88	0	0.77 ± 0.06	0.76 ± 0.06	−0.011 ± 0.012	11 (7.2)	0	140.8 (111.2, 188)
17	Zolotaya	68.8663	37.0166	Parr/fry	89	70	228	10.21	0	0.76 ± 0.06	0.77 ± 0.06	−0.005 ± 0.012	12 (7.8)	2 (11.1)	192.1 (149.2, 264.8)
18	Rynda	68.9333	36.8500	Parr/fry	79	75	242	10.51	1	0.78 ± 0.06	0.77 ± 0.06	−0.023 ± 0.006	19 (12.4)	1 (5.6)	447.5 (308.1, 793.4)
19	Orlovka	69.2043	35.2920	Parr/fry	69	35	171	8.75	0	0.75 ± 0.06	0.74 ± 0.06	−0.027 ± 0.017	15 (9.8)	1 (5.6)	31.2 (27.1, 36.4)
20	Dolgaya	69.1500	34.9333	Parr/fry	76	44	191	9.25	0	0.77 ± 0.06	0.75 ± 0.06	−0.033 ± 0.02	14 (9.2)	3 (16.7)	47.3 (41.1, 55.3)
21	Kola	68.8833	33.0333	Parr/fry	97	94	244	10.28	0	0.77 ± 0.06	0.76 ± 0.06	−0.009 ± 0.009	10 (6.5)	3 (16.7)	296.8 (229.7, 412.3)
22	Pak	68.7667	32.4167	Parr/fry	92	73	206	9.46	0	0.75 ± 0.06	0.75 ± 0.06	−0.002 ± 0.011	23 (15.0)	5 (27.8)	258.6 (197.4, 368)
23	Ulita	68.6833	32.1000	Parr/fry	74	60	197	8.88	0	0.70 ± 0.05	0.73 ± 0.05	0.030 ± 0.016	4 (2.6)	3 (16.7)	74.5 (63.7, 88.6)
24	Pecha	68.5833	31.8000	Parr/fry	66	56	206	9.63	1	0.73 ± 0.06	0.75 ± 0.06	0.019 ± 0.013	7 (4.6)	1 (5.6)	471.2 (256, 2,362.9)
25	Kulonga	69.0785	33.1292	Parr/fry	47	36	143	7.24	0	0.71 ± 0.06	0.70 ± 0.06	−0.039 ± 0.021	10 (6.5)	1 (5.6)	67.8 (50.9, 97.5)
26	Ura	69.2833	32.8167	Parr/fry	103	73	228	10.09	0	0.75 ± 0.05	0.75 ± 0.06	−0.002 ± 0.018	18 (11.8)	2 (11.1)	112.9 (98.3, 131.6)
27	B. Zap. Litsa	69.4171	32.2021	Parr/fry	99	69	216	9.86	0	0.74 ± 0.06	0.76 ± 0.06	0.010 ± 0.009	13 (8.5)	1 (5.6)	168.8 (138.2, 214.4)
28	Titovka	69.5167	31.9667	Parr/fry	91	80	250	10.70	0	0.76 ± 0.06	0.77 ± 0.06	0.007 ± 0.014	6 (3.9)	4 (22.2)	151.3 (125.9, 187.2)
29	Pyave	69.7939	32.5119	Parr/fry	38	28	167	8.76	0	0.70 ± 0.05	0.72 ± 0.05	0.002 ± 0.019	4 (2.6)	1 (5.6)	61.8 (44, 98.7)
30	Pechenga	69.5500	31.2500	Parr/fry	44	33	182	9.34	1	0.75 ± 0.06	0.75 ± 0.06	−0.014 ± 0.018	9 (5.9)	0	151.4 (100.6, 288.9)
31	Grense Jakobselv	69.7782	30.8383	Parr	80	55	222	10.34	0	0.76 ± 0.06	0.76 ± 0.06	−0.021 ± 0.011	10 (6.5)	2 (11.1)	139.3 (112.7, 179.8)
32	Karpelva	69.6672	30.3849	Parr	92	68	214	9.76	0	0.75 ± 0.06	0.75 ± 0.06	−0.017 ± 0.018	9 (5.9)	2 (11.1)	192.6 (151.3, 260.7)
33	Munkelva	69.6489	29.4597	Parr	93	86	220	9.68	0	0.75 ± 0.06	0.75 ± 0.06	−0.001 ± 0.018	5 (3.3)	1 (5.6)	315.4 (228.8, 493.2)
34	Neiden	69.7006	29.5208	Parr	94	72	227	10.20	0	0.76 ± 0.06	0.75 ± 0.06	−0.017 ± 0.013	17 (11.1)	3 (16.7)	255.7 (196.6, 359.3)
35	Klokkarelva	69.8580	29.3867	Parr	94	88	223	9.75	0	0.75 ± 0.06	0.75 ± 0.06	0.000 ± 0.018	10 (6.5)	1 (5.6)	514.4 (331, 1,093.4)
36	Vesterelva	70.1587	28.5804	Parr	93	83	220	9.47	0	0.74 ± 0.06	0.74 ± 0.06	−0.009 ± 0.013	5 (3.3)	1 (5.6)	126.7 (105.5, 156.4)
37	Bergebyelva	70.1503	28.8985	Parr	107	87	206	9.07	0	0.77 ± 0.05	0.76 ± 0.05	−0.028 ± 0.013	6 (3.9)	0	185 (152.4, 232.4)
38	Vestre Jakobselv	70.1092	29.3285	Parr	78	72	215	9.49	0	0.77 ± 0.05	0.75 ± 0.05	−0.041 ± 0.014	8 (5.2)	2 (11.1)	149.7 (118.6, 199.2)
39	Skallelva	70.1856	30.3284	Parr	94	90	214	9.24	1	0.73 ± 0.06	0.75 ± 0.06	0.011 ± 0.010	11 (7.2)	2 (11.1)	258.1 (197.6, 364.7)
40	Komagelva	70.2422	30.5232	Parr	94	78	192	8.61	0	0.71 ± 0.06	0.70 ± 0.06	−0.016 ± 0.013	10 (6.5)	0	248.8 (170.3, 437.5)
41	Syltefjordelva	70.5311	30.0115	Parr	93	83	193	8.18	0	0.70 ± 0.06	0.71 ± 0.05	0.021 ± 0.018	15 (9.8)	3 (16.7)	167.8 (129.4, 232.8)
42	Kongsfjordelva	70.6570	29.2569	Parr	91	82	204	8.88	0	0.72 ± 0.06	0.73 ± 0.06	0.011 ± 0.017	15 (9.8)	1 (5.6)	280.2 (193.1, 486.8)
43	Tana‐Iesjohka	69.4200	24.7499	Parr	94	78	217	9.52	0	0.76 ± 0.07	0.73 ± 0.07	−0.021 ± 0.018	10 (6.5)	2 (11.1)	291 (205.2, 482.2)
44	Tana‐Laksjohka	70.0651	27.5524	Parr	92	83	235	9.86	0	0.72 ± 0.06	0.73 ± 0.06	0.008 ± 0.010	15 (9.8)	3 (16.7)	667.9 (389.4, 2,119.1)
45	Langfjordelva	70.6179	27.6089	Parr	93	65	217	9.34	0	0.74 ± 0.05	0.75 ± 0.05	0.007 ± 0.013	13 (8.5)	4 (22.2)	79.2 (66.7, 96.1)
46	Børselva	70.3121	25.5208	Parr	92	83	236	9.98	0	0.76 ± 0.06	0.75 ± 0.06	−0.014 ± 0.019	15 (9.8)	5 (27.8)	174.6 (141.9, 223.7)
47	Lakselva	70.0825	24.9202	Parr	94	90	211	8.83	0	0.72 ± 0.06	0.71 ± 0.06	−0.012 ± 0.013	10 (6.5)	3 (16.7)	1,155.9 (461.1, Infinite)
48	Stabburselva	70.1846	24.9336	Parr	89	79	207	9.18	0	0.74 ± 0.06	0.72 ± 0.06	−0.032 ± 0.014	13 (8.5)	1 (5.6)	185.7 (137.1, 278.1)
49	Repparfjordselva	70.4480	24.3223	Parr	92	90	247	10.23	2	0.76 ± 0.06	0.75 ± 0.06	−0.028 ± 0.015	11 (7.2)	3 (16.7)	417.8 (292.5, 708.1)
50	Alta	69.9691	23.3752	Parr	85	84	220	9.40	1	0.79 ± 0.06	0.73 ± 0.06	−0.002 ± 0.011	7 (4.6)	1 (5.6)	2,941.8 (659.4, Infinite)
51	Reisa	69.7837	21.0095	Parr	64	61	196	9.02	0	0.71 ± 0.06	0.72 ± 0.06	0.001 ± 0.015	10 (6.5)	2 (11.1)	843.9 (293.9, Infinite)
52	Målselv	69.2744	18.5146	Parr	82	77	227	9.92	1	0.73 ± 0.06	0.76 ± 0.06	0.032 ± 0.017	5 (3.3)	1 (5.6)	3,147.2 (469.8, Infinite)
53	Laukhelle	69.2287	17.8531	Parr	87	72	232	10.09	0	0.77 ± 0.06	0.76 ± 0.06	−0.026 ± 0.012	10 (6.5)	1 (5.6)	432.6 (289.5, 823.8)
54	Roksdalsvassdraget	69.0502	15.8692	Parr	60	58	207	9.43	0	0.75 ± 0.05	0.75 ± 0.05	−0.006 ± 0.014	5 (3.3)	1 (5.6)	140.6 (110, 190.9)
55	Alvsvågvassdraget	68.9168	15.2343	Parr	57	45	206	9.85	0	0.79 ± 0.05	0.77 ± 0.05	−0.040 ± 0.019	11 (7.2)	3 (16.7)	92.1 (75.8, 115.9)
56	Gårdselva	68.8300	15.6572	Parr	104	88	241	10.14	0	0.78 ± 0.05	0.77 ± 0.05	−0.017 ± 0.011	7 (4.6)	2 (11.1)	315.5 (228.6, 494.8)
57	Saltdalselva	67.1023	15.4224	Parr	71	52	218	10.18	0	0.78 ± 0.05	0.77 ± 0.05	−0.033 ± 0.014	9 (5.9)	1 (5.6)	291.2 (190.3, 590)
58	Beiarelva	67.0302	14.5743	Parr	70	69	222	10.05	1	0.77 ± 0.06	0.76 ± 0.06	−0.013 ± 0.014	9 (5.9)	1 (5.6)	139.2 (194.8, Infinite)
59	Åbjøra	65.0784	12.4556	Parr	44	88	248	10.47	0	0.76 ± 0.05	0.78 ± 0.05	0.007 ± 0.017	13 (8.5)	1 (5.6)	101.2 (124.1, Infinite)
60	Namsen	64.4657	11.5448	Parr	91	85	244	10.22	0	0.75 ± 0.05	0.76 ± 0.05	0.001 ± 0.009	10 (6.5)	2 (11.1)	583 (327.8, 2,229.2)
61	Bogna	64.3880	11.3947	Parr	104	97	240	10.07	0	0.77 ± 0.05	0.77 ± 0.05	−0.011 ± 0.011	13 (8.5)	3 (16.7)	696.6 (423.6, 1816.6)
62	Årgårdsvassdraget	64.3127	11.2237	Parr	94	87	236	10.12	2	0.76 ± 0.05	0.77 ± 0.05	0.012 ± 0.014	10 (6.5)	6 (33.3)	959.8 (429.7, Infinite)
63	Steinsdalselva	64.2977	10.5034	Parr	93	70	228	10.07	0	0.75 ± 0.06	0.76 ± 0.05	0.008 ± 0.017	6 (3.9)	1 (5.6)	298.2 (213, 483.1)
64	Nordelva	63.9615	10.2221	Parr	34	33	199	10.03	0	0.75 ± 0.06	0.75 ± 0.05	−0.019 ± 0.018	3 (2.0)	1 (5.6)	719.5 (759.9, Infinite)
65	Stordalselva	63.9594	10.2269	Parr	68	65	226	10.17	0	0.77 ± 0.06	0.76 ± 0.06	−0.023 ± 0.014	9 (5.9)	2 (11.1)	2,494.4 (655, Infinite)
66	Skauga	63.5931	9.9195	Parr	71	63	219	9.87	0	0.77 ± 0.06	0.77 ± 0.05	−0.008 ± 0.016	13 (8.5)	1 (5.6)	403 (251.2, 950.1)
67	Verdalselva	63.8033	11.4591	Parr	95	79	233	10.03	1	0.76 ± 0.06	0.75 ± 0.06	−0.015 ± 0.010	11 (7.2)	1 (5.6)	400.2 (267, 764)
68	Levangerelva	63.7530	11.2990	Parr	83	75	202	9.08	0	0.76 ± 0.05	0.76 ± 0.05	−0.013 ± 0.011	8 (5.2)	4 (22.2)	301.5 (220.8, 463.9)
69	Stjørdalselva	63.4489	10.9039	Parr	92	84	239	10.33	0	0.77 ± 0.05	0.77 ± 0.05	−0.013 ± 0.008	13 (8.5)	1 (5.6)	594.6 (364.4, 1504.6)
70	Homla	63.4145	10.8023	Parr	93	87	222	9.56	0	0.75 ± 0.05	0.75 ± 0.05	−0.012 ± 0.012	10 (6.5)	0	314.1 (230.6, 479.4)
71	Nidelva	63.4431	10.4146	Parr	82	73	234	10.14	0	0.76 ± 0.06	0.75 ± 0.06	−0.021 ± 0.017	8 (5.2)	1 (5.6)	330.2 (231, 560.4)
72	Gaula	63.3429	10.2286	Parr	93	90	244	10.25	0	0.77 ± 0.06	0.77 ± 0.05	−0.010 ± 0.014	6 (3.9)	1 (5.6)	2,508.1 (672.2, Infinite)
73	Vigda	63.3124	10.1824	Parr	85	82	213	9.20	0	0.74 ± 0.06	0.75 ± 0.06	0.014 ± 0.014	7 (4.6)	2 (11.1)	1,142.1 (494.8, Infinite)
74	Børsa	63.3252	10.0759	Parr	46	40	191	9.47	0	0.74 ± 0.06	0.75 ± 0.05	0.008 ± 0.017	12 (7.8)	1 (5.6)	416.4 (206.1, 13,513.3)
75	Orkla	63.3101	9.8303	Parr	104	101	253	10.27	0	0.76 ± 0.06	0.76 ± 0.05	−0.003 ± 0.010	13 (8.5)	2 (11.1)	1,328.8 (633, Infinite)
76	Surna	62.9706	8.6501	Parr	79	79	236	10.13	0	0.76 ± 0.06	0.77 ± 0.05	0.002 ± 0.010	18 (11.8)	3 (16.7)	2,769 (648.7, Infinite)
77	Eiravassdraget	62.6851	8.1306	Parr	81	73	230	10.16	0	0.78 ± 0.06	0.77 ± 0.06	−0.021 ± 0.009	17 (11.1)	1 (5.6)	162.5 (133.9, 204.3)
78	Visa	62.7229	7.9266	Parr	36	35	194	9.92	0	0.75 ± 0.05	0.77 ± 0.05	0.008 ± 0.016	10 (6.5)	0	128.6 (87.7, 229.2)
79	Korsbrekkeelva	62.0818	6.8758	Parr	45	44	220	10.40	0	0.76 ± 0.06	0.77 ± 0.06	0.014 ± 0.016	5 (3.3)	3 (16.7)	234.8 (162.6, 409.1)
80	Ørstaelva	62.1959	6.1241	Adult	36	34	207	10.45	0	0.77 ± 0.05	0.78 ± 0.05	−0.009 ± 0.019	14 (9.2)	0	3,016.6 (371, Infinite)
81	Ervikelva	62.1648	5.1103	Parr	61	60	218	10.17	0	0.77 ± 0.05	0.79 ± 0.05	0.010 ± 0.010	11 (7.2)	1 (5.6)	573 (296.7, 5,108.4)
82	Eidselva	61.9020	5.9846	Adult	104	103	259	10.35	0	0.75 ± 0.05	0.77 ± 0.05	0.014 ± 0.008	9 (5.9)	5 (27.8)	670.3 (414.9, 1626.9)
83	Stryneelva	61.9018	6.7172	Parr	73	68	225	10.08	1	0.78 ± 0.05	0.78 ± 0.05	−0.013 ± 0.017	5 (3.3)	0	800.1 (401.5, 16,315)
84	Gjengedalsvassdraget	61.7328	5.9173	Parr	63	58	225	10.23	0	0.76 ± 0.05	0.77 ± 0.05	0.011 ± 0.014	16 (10.5)	3 (16.7)	449.2 (10,713.6, Infinite)
85	Osenelva	61.5512	5.3997	Parr	94	77	222	9.80	0	0.78 ± 0.05	0.77 ± 0.05	−0.026 ± 0.015	14 (9.2)	1 (5.6)	296 (217, 454.6)
86	Nausta	61.5061	5.7198	Parr	74	73	231	10.29	0	0.76 ± 0.06	0.78 ± 0.05	0.018 ± 0.017	11 (7.2)	3 (16.7)	1,315.7 (434.2, Infinite)
87	Jølstra	61.4581	5.8306	Parr	91	84	255	10.70	0	0.78 ± 0.05	0.78 ± 0.05	−0.002 ± 0.010	7 (4.6)	1 (5.6)	373 (260.8, 632.5)
88	Gaula, Sunnfjord	61.3681	5.6739	Parr	63	60	216	10.09	0	0.75 ± 0.05	0.76 ± 0.05	0.010 ± 0.009	16 (10.5)	1 (5.6)	565.5 (1503.7, Infinite)
89	Flekkeelva	61.3112	5.3452	Parr	93	90	188	8.16	0	0.72 ± 0.05	0.72 ± 0.05	−0.002 ± 0.011	12 (7.8)	4 (22.2)	577.9 (319.6, 2,422.4)
90	Daleelva,Høyanger	61.2158	6.0731	Adult	104	98	252	10.36	0	0.76 ± 0.05	0.78 ± 0.05	0.013 ± 0.007	9 (5.9)	4 (22.2)	504 (338.8, 944.3)
91	Årøyelva	61.2685	7.1664	Adult	102	81	213	9.78	1	0.75 ± 0.06	0.76 ± 0.06	0.006 ± 0.014	14 (9.2)	4 (22.2)	170.9 (620, Infinite)
92	Flåmselva	60.8649	7.1191	Parr	81	74	217	9.52	0	0.76 ± 0.05	0.75 ± 0.05	−0.025 ± 0.012	19 (12.4)	1 (5.6)	269.7 (167.7, 626.5)
93	Nærøydalselva	60.8808	6.8430	Adult	68	57	200	9.46	0	0.76 ± 0.06	0.76 ± 0.05	−0.017 ± 0.016	5 (3.3)	2 (11.1)	162.1 (120.8, 239.4)
94	Vikja	61.0897	6.5857	Parr	64	64	244	10.67	0	0.78 ± 0.05	0.78 ± 0.05	0.001 ± 0.010	4 (2.6)	1 (5.6)	3,062.8 (630.4, Infinite)
95	Loneelva	60.5257	5.4895	Parr	85	79	213	9.45	1	0.77 ± 0.05	0.77 ± 0.05	−0.008 ± 0.017	7 (4.6)	2 (11.1)	576 (330.9, 1965.6)
96	Oselva	60.1836	5.4707	Parr	92	84	227	9.93	1	0.76 ± 0.05	0.77 ± 0.05	0.003 ± 0.017	21 (13.7)	1 (5.6)	206.2 (164, 273.1)
97	Etneelva	59.6730	5.9342	Parr	93	83	233	10.00	0	0.77 ± 0.06	0.76 ± 0.06	−0.012 ± 0.010	11 (7.2)	2 (11.1)	267.8 (192.2, 426.3)
98	Vikedalselva	59.4969	5.8971	Parr	92	82	222	9.87	0	0.77 ± 0.06	0.76 ± 0.05	−0.010 ± 0.011	14 (9.2)	2 (11.1)	189.6 (153.9, 243.4)
99	Suldalslågen	59.4811	6.2488	Parr	91	86	227	9.86	1	0.74 ± 0.05	0.75 ± 0.05	0.000 ± 0.016	11 (7.2)	1 (5.6)	295.3 (223.2, 427.7)
100	Vormo	59.2717	6.3326	Parr	94	94	238	10.16	0	0.78 ± 0.06	0.77 ± 0.05	−0.014 ± 0.013	8 (5.2)	5 (27.8)	128.1 (111, 150.2)
101	Årdalselva	59.1438	6.1694	Parr	93	89	240	10.34	0	0.77 ± 0.05	0.77 ± 0.05	0.004 ± 0.011	18 (11.8)	0	395.7 (279.2, 658.1)
102	Lyseelva	59.0517	6.6452	Parr	94	67	225	10.12	0	0.76 ± 0.05	0.76 ± 0.05	−0.015 ± 0.009	13 (8.5)	1 (5.6)	130.9 (107.7, 164.6)
103	Espedalsvassdraget	58.8602	6.1504	Parr	94	84	236	10.41	0	0.75 ± 0.05	0.77 ± 0.05	0.019 ± 0.010	8 (5.2)	5 (27.8)	325.5 (234.7, 515.8)
104	Frafjordselva	58.8435	6.2799	Parr	87	76	245	10.64	0	0.77 ± 0.06	0.77 ± 0.05	−0.001 ± 0.017	6 (3.9)	0	466.8 (303.7, 961.1)
105	Figgjo	58.8100	5.5468	Parr	93	89	247	10.60	0	0.77 ± 0.05	0.78 ± 0.05	−0.004 ± 0.015	10 (6.5)	2 (11.1)	662.4 (401.5, 1756.5)
106	Håelva	58.6695	5.5438	Parr	63	61	232	10.32	3	0.77 ± 0.06	0.77 ± 0.05	−0.020 ± 0.009	7 (4.6)	1 (5.6)	544.9 (305.9, 2,162.8)
107	Ogna	58.5169	5.7920	Parr	60	56	222	10.06	1	0.77 ± 0.05	0.77 ± 0.05	−0.019 ± 0.014	12 (7.8)	3 (16.7)	147.2 (113.8, 204)
108	Bjerkreimselva	58.4779	5.9949	Parr	83	83	219	9.62	0	0.77 ± 0.06	0.76 ± 0.05	−0.016 ± 0.010	11 (7.2)	3 (16.7)	1917 (1,440.2, Infinite)
109	Soknedalselva	58.3214	6.2857	Parr	24	24	190	10.46	1	0.79 ± 0.05	0.78 ± 0.05	−0.044 ± 0.020	3 (2.0)		679.8 (570.8, Infinite)
110	Kvina	58.2730	6.8910	Parr	91	90	240	10.14	1	0.77 ± 0.05	0.77 ± 0.05	−0.005 ± 0.012	19 (12.4)	2 (11.1)	407.9 (289.6, 670.3)
111	Mandalselva	58.0203	7.4563	Parr	45	43	211	10.12	0	0.76 ± 0.06	0.765 ± 0.06	−0.006 ± 0.011	8 (5.2)	1 (5.6)	814.1 (321, Infinite)
112	Otra	58.1441	8.0132	Parr	76	71	246	10.76	1	0.78 ± 0.06	0.78 ± 0.05	−0.007 ± 0.017	8 (5.2)	4 (22.2)	958.5 (470.1, 4,113,951.3)
113	Storelva	58.7607	9.0766	Smolt	79	75	229	10.10	0	0.75 ± 0.05	0.76 ± 0.06	−0.006 ± 0.018	12 (7.8)	4 (22.2)	242.6 (181.4, 357.5)
114	Numedalslågen	59.0378	10.0553	Parr	84	83	238	10.10	4	0.73 ± 0.05	0.75 ± 0.05	0.013 ± 0.020	10 (6.5)	5 (27.8)	3,001 (695.8, Infinite)
115	Ennigdalselva	58.9818	11.4746	Adult	94	86	192	8.21	2	0.71 ± 0.05	0.72 ± 0.06	0.005 ± 0.020	13 (8.5)	2 (11.1)	368.4 (221.1, 973.4)
