# Supplementary material for: Population genetic analysis reveals a geographically limited transition zone between two genetically distinct Atlantic salmon lineages in Norway
Source: Ecol Evol. 2019 May 22;9(12):6901–21. doi: 10.1002/ece3.5258 (PMC6662299; doi:10.1002/ece3.5258)
Supplement: Supplementary file 3 [file ECE3-9-6901-s003.docx]

| 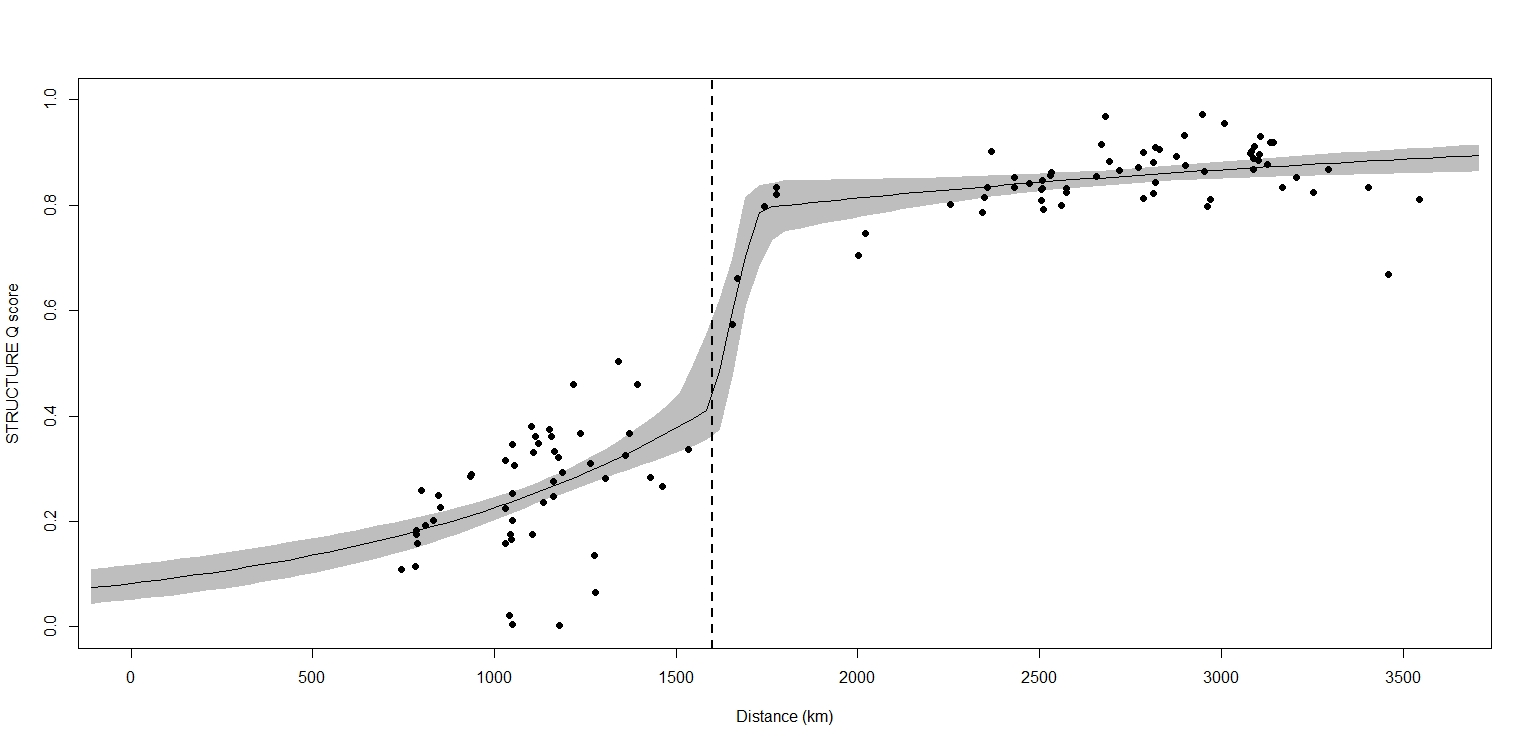 | 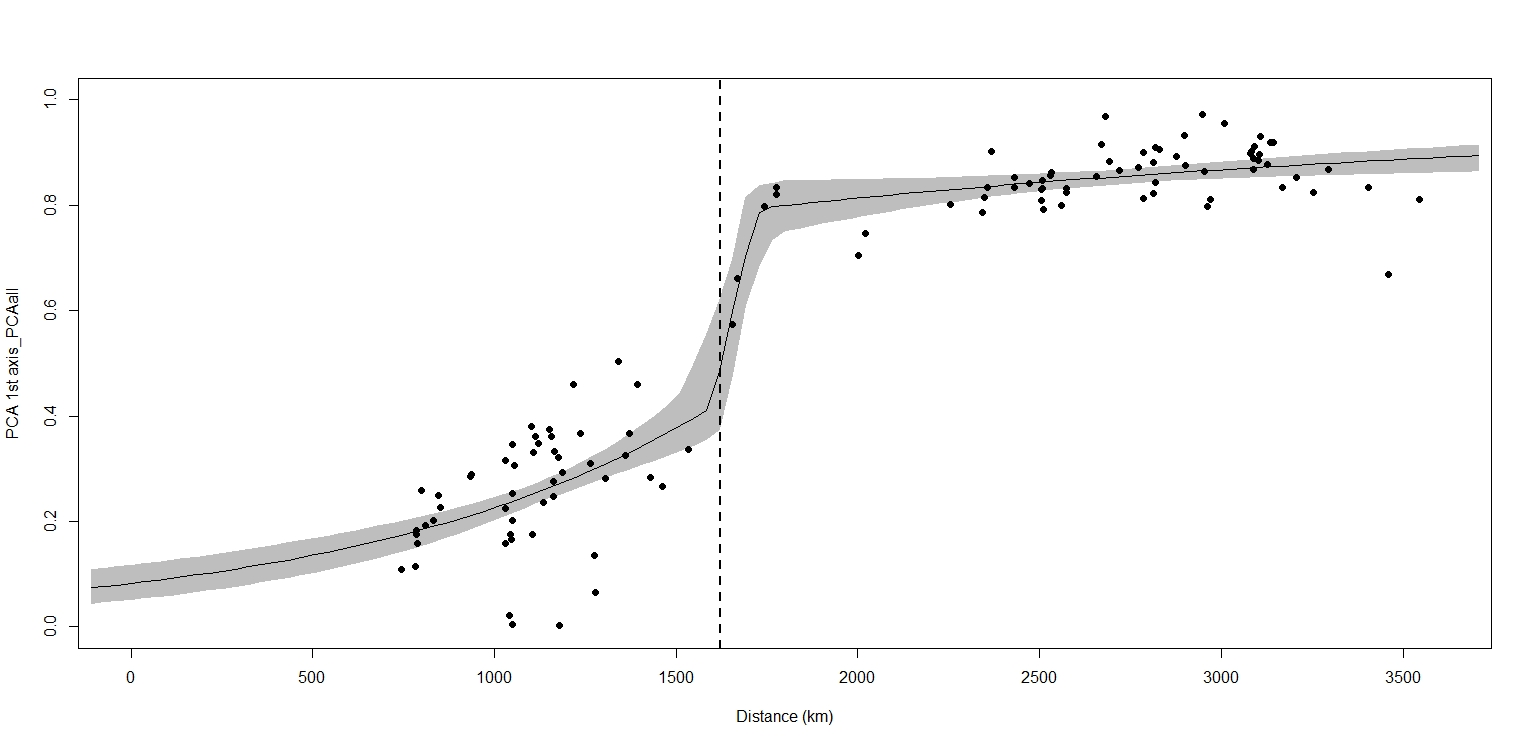 |
| --- | --- |
| 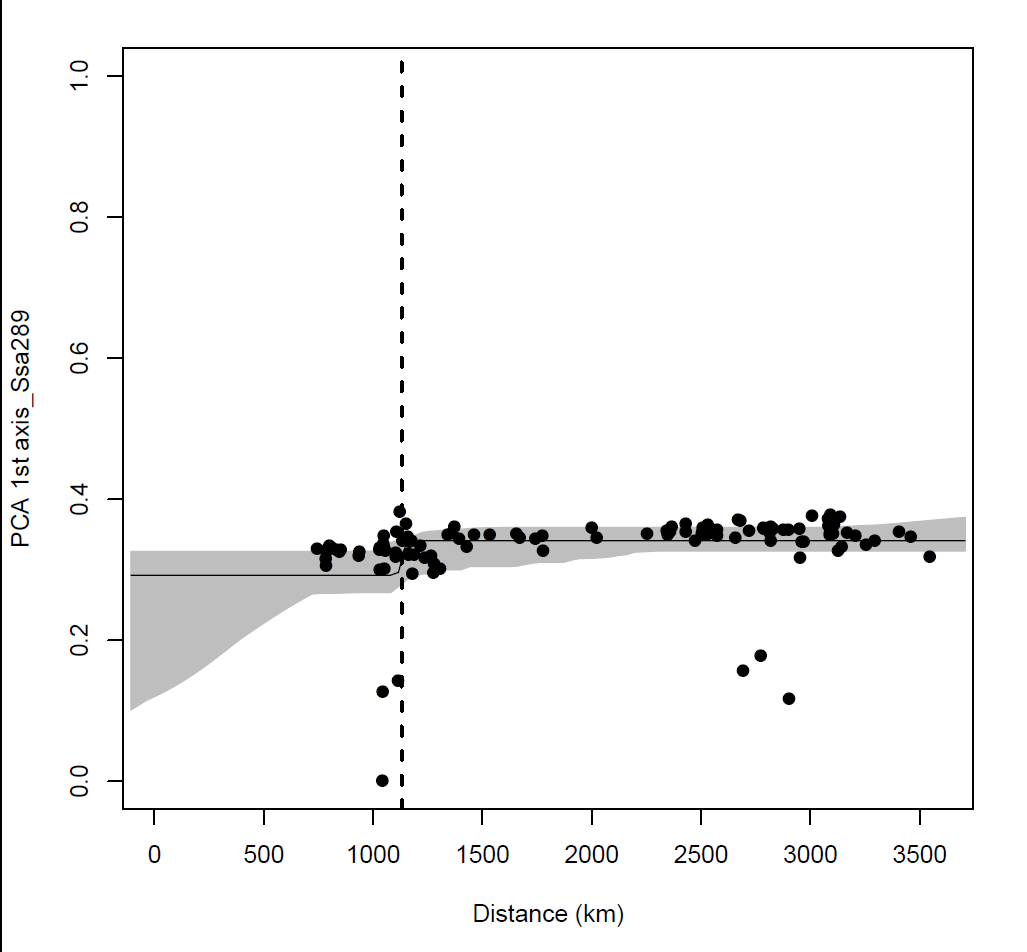 | 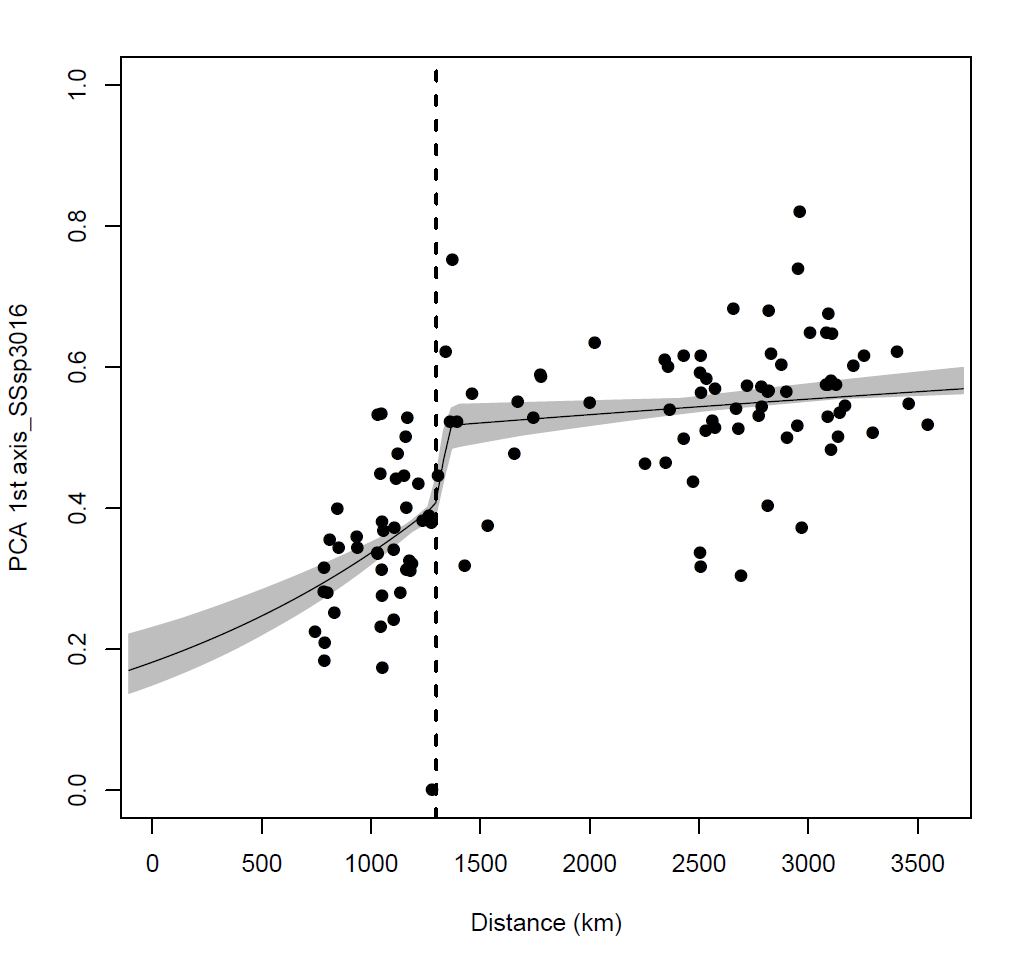 |
| 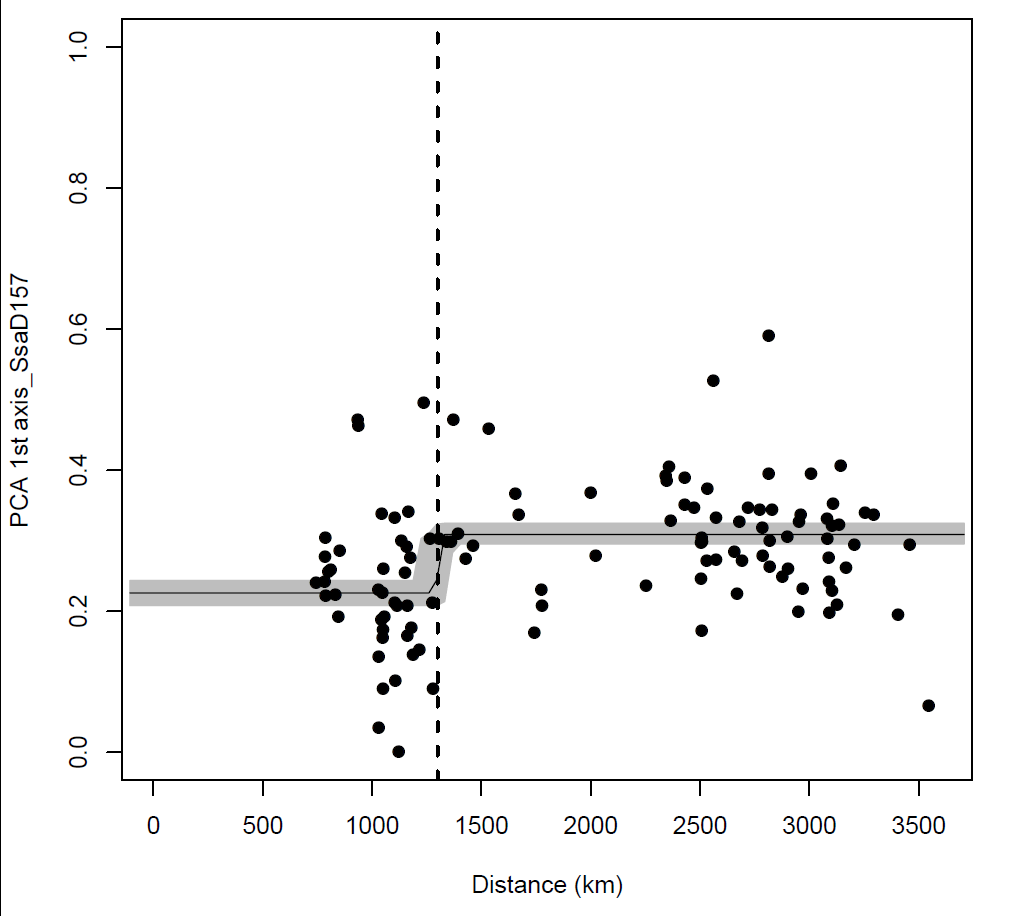 | 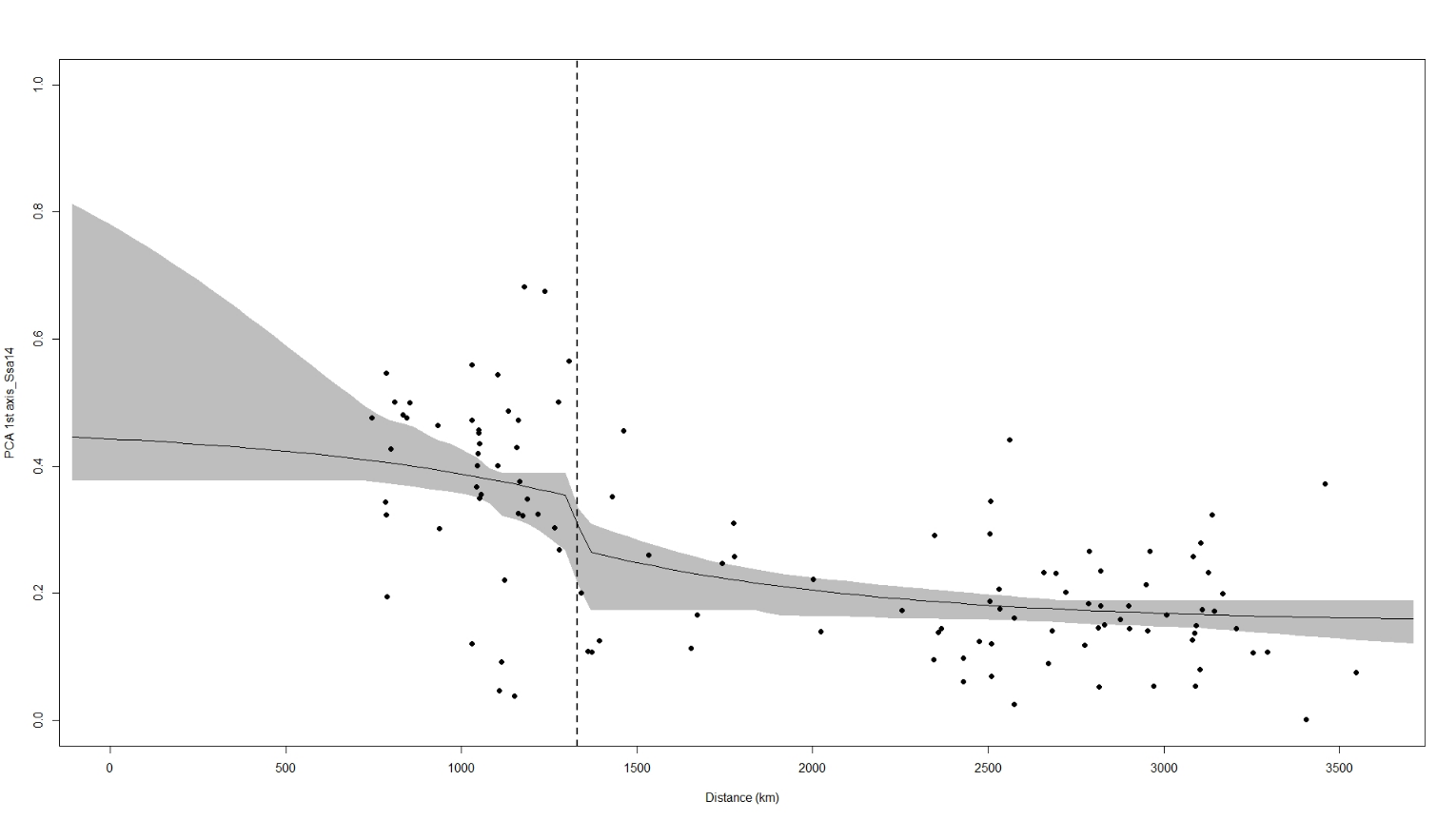 |
| 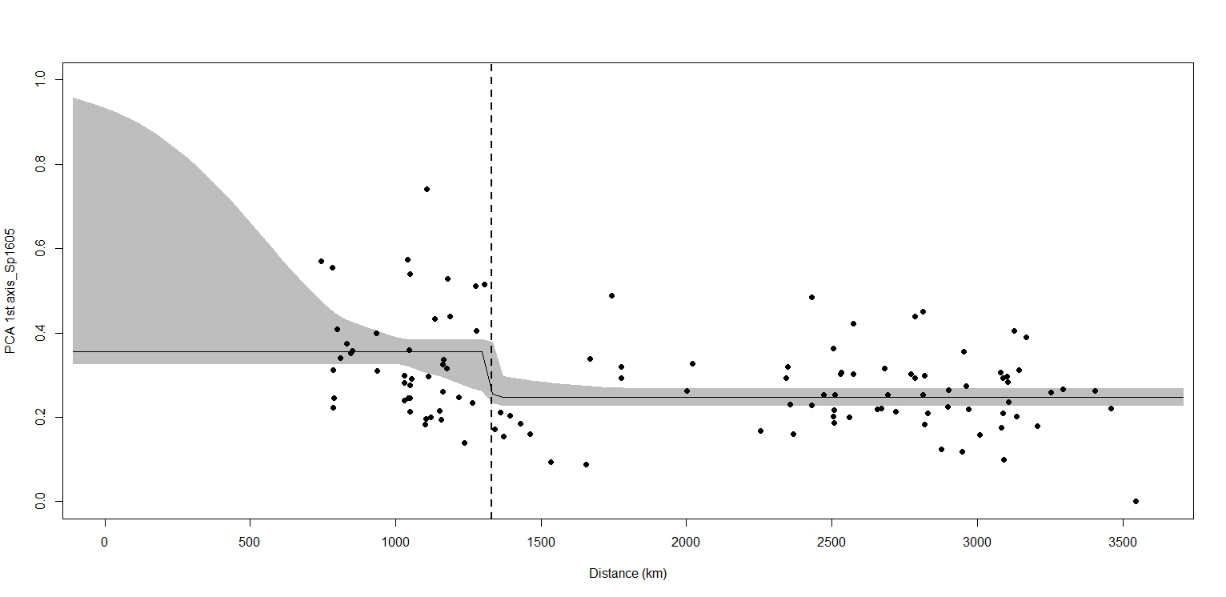 | 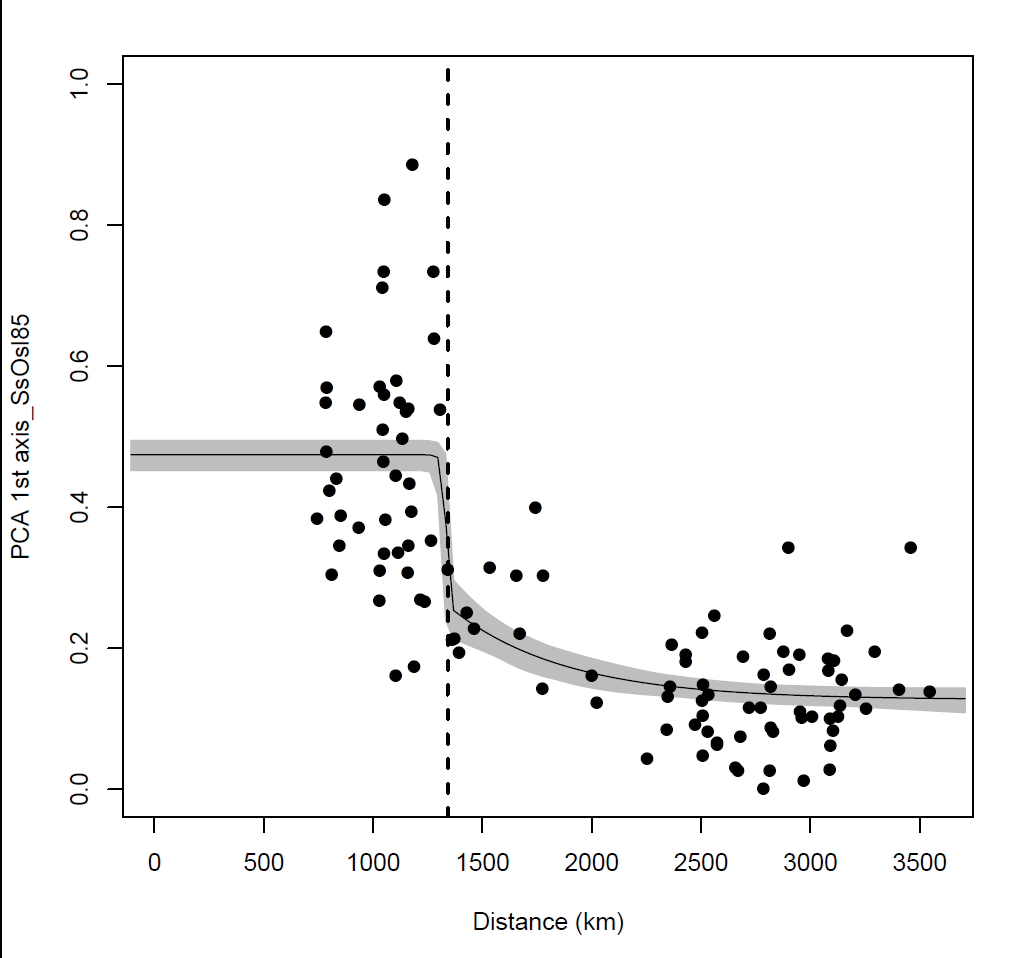 |
| 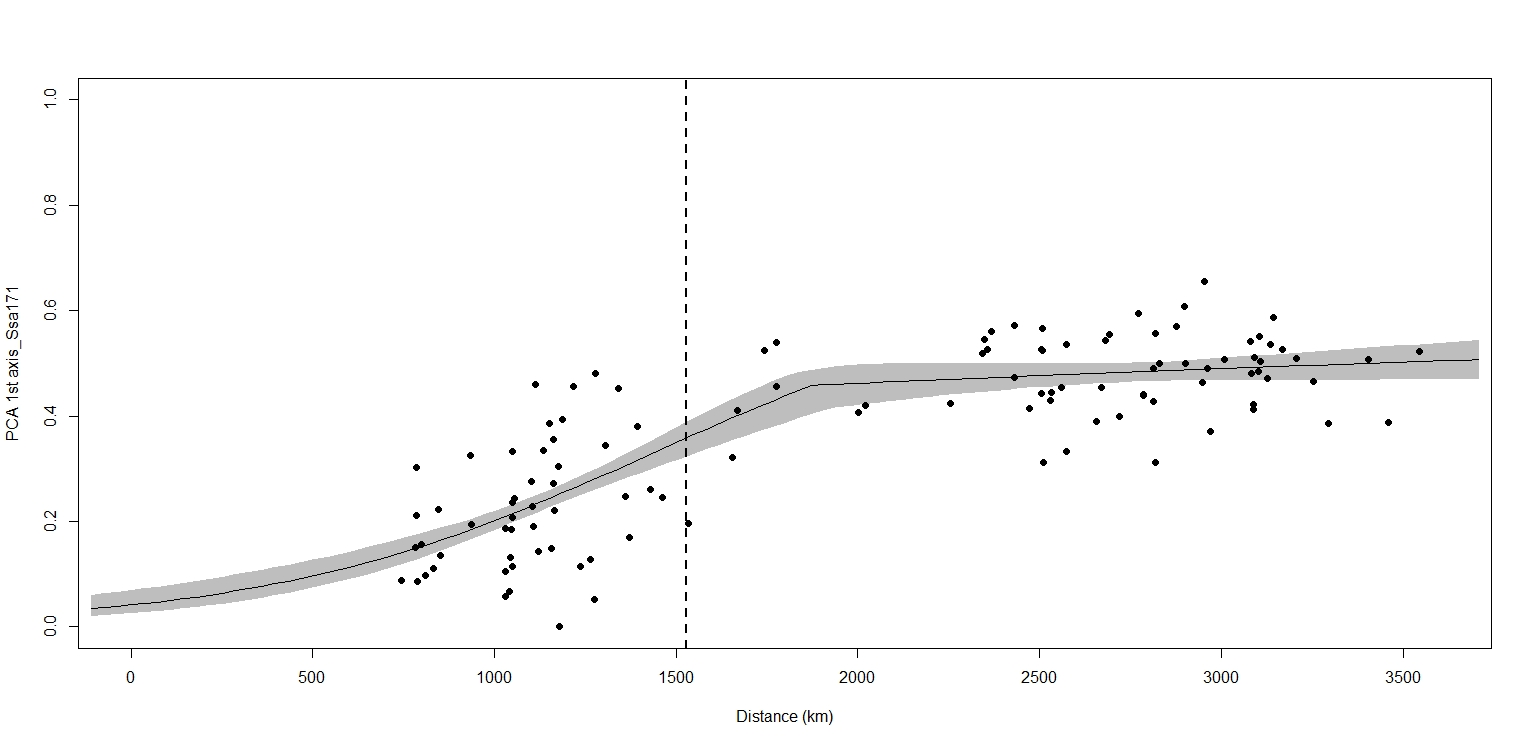 | 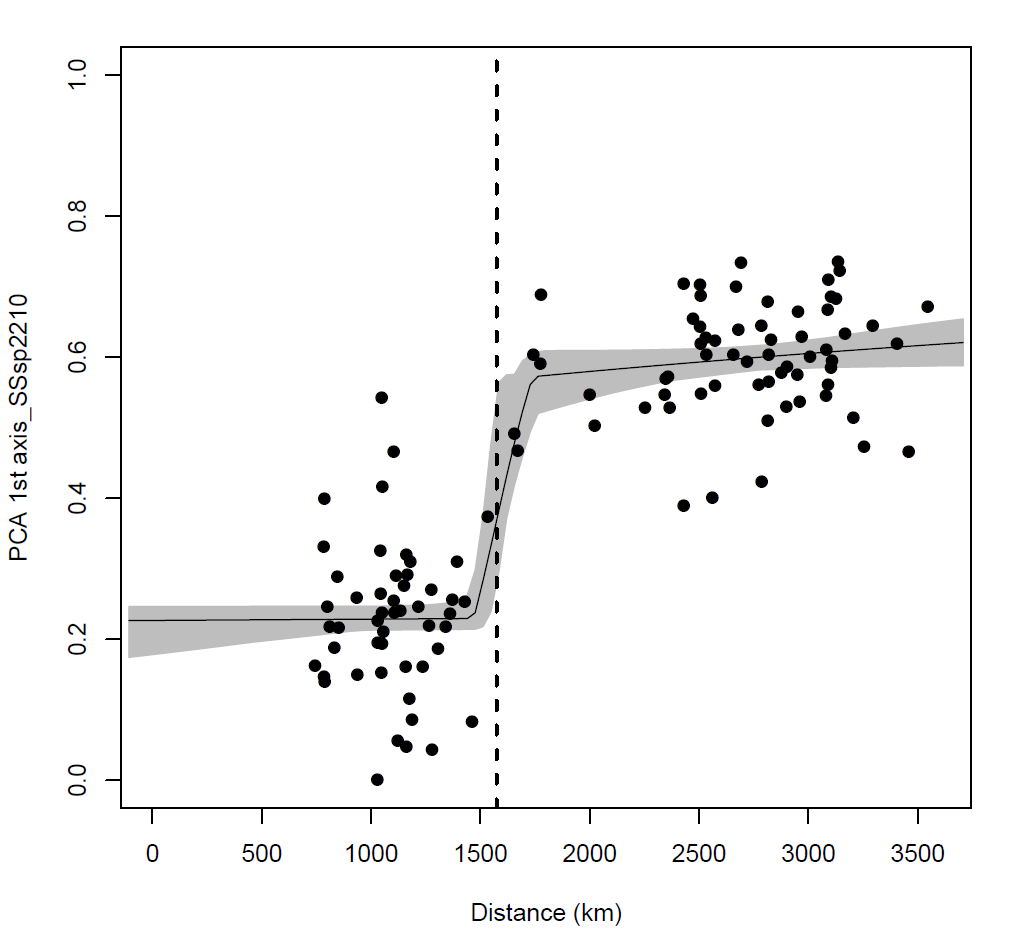 |
| 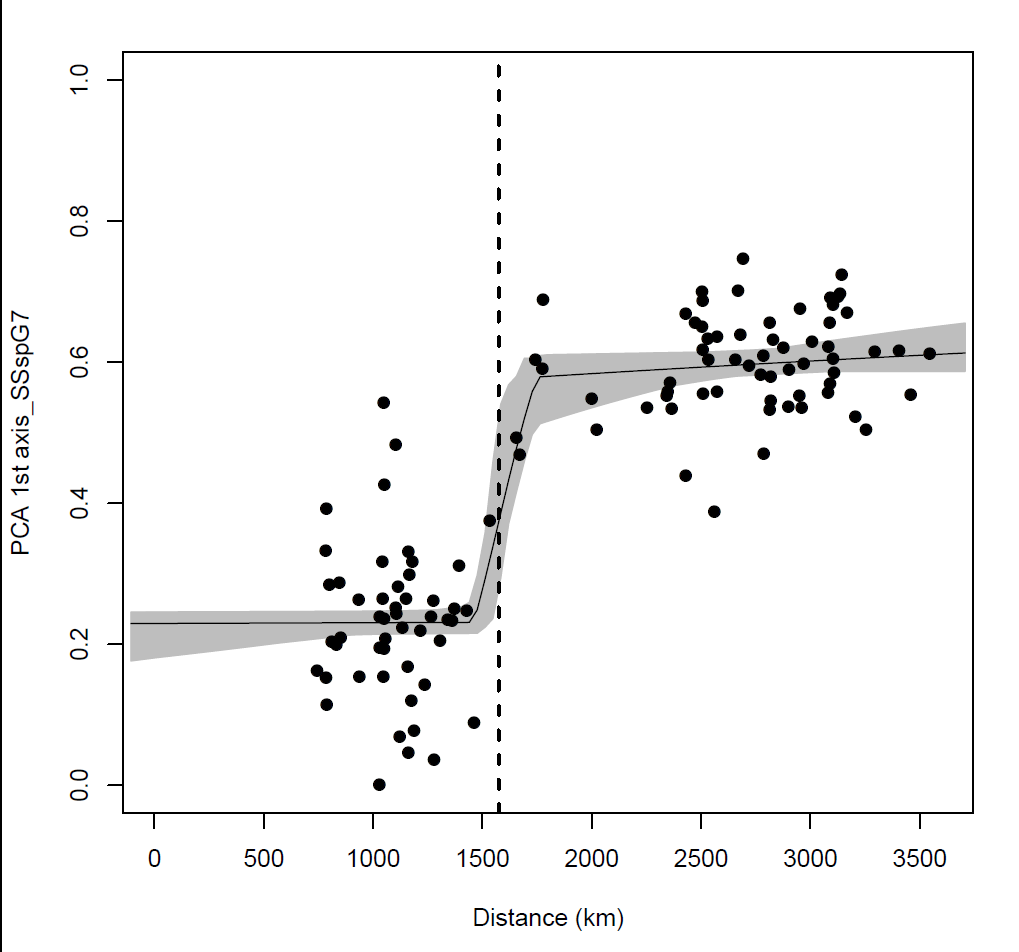 | 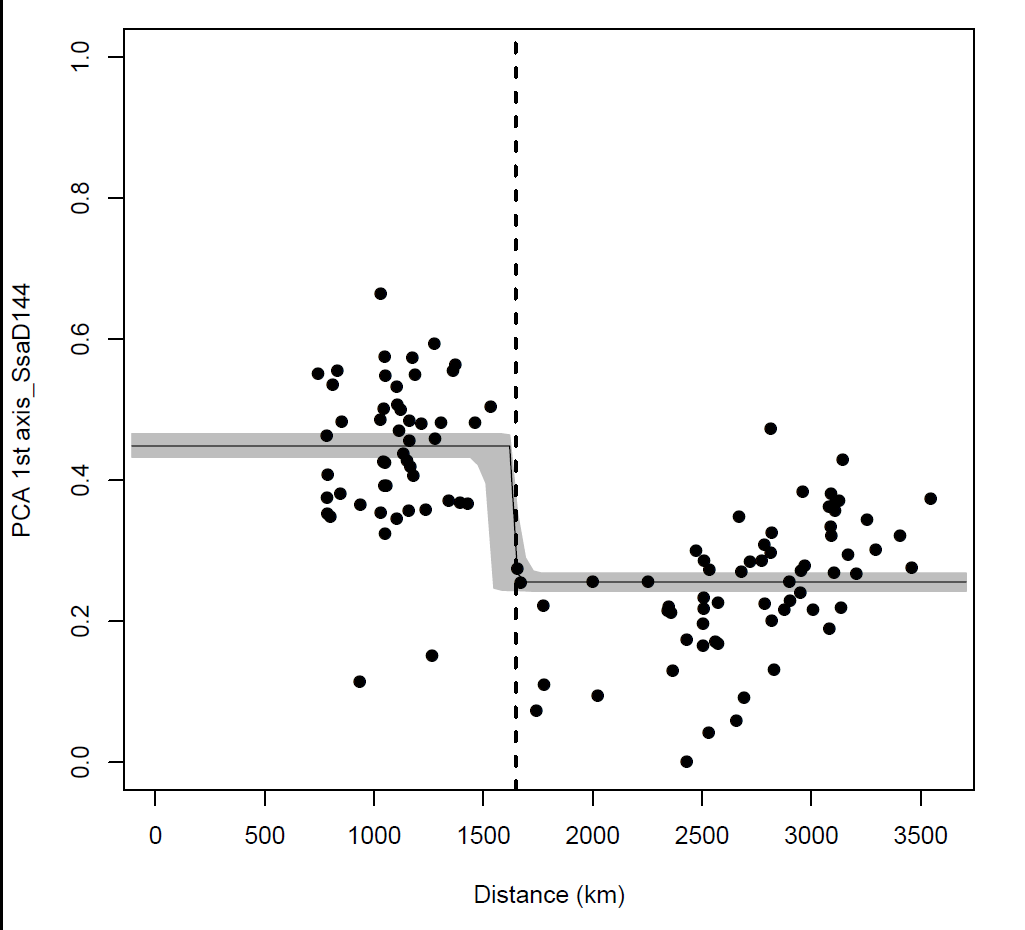 |
| 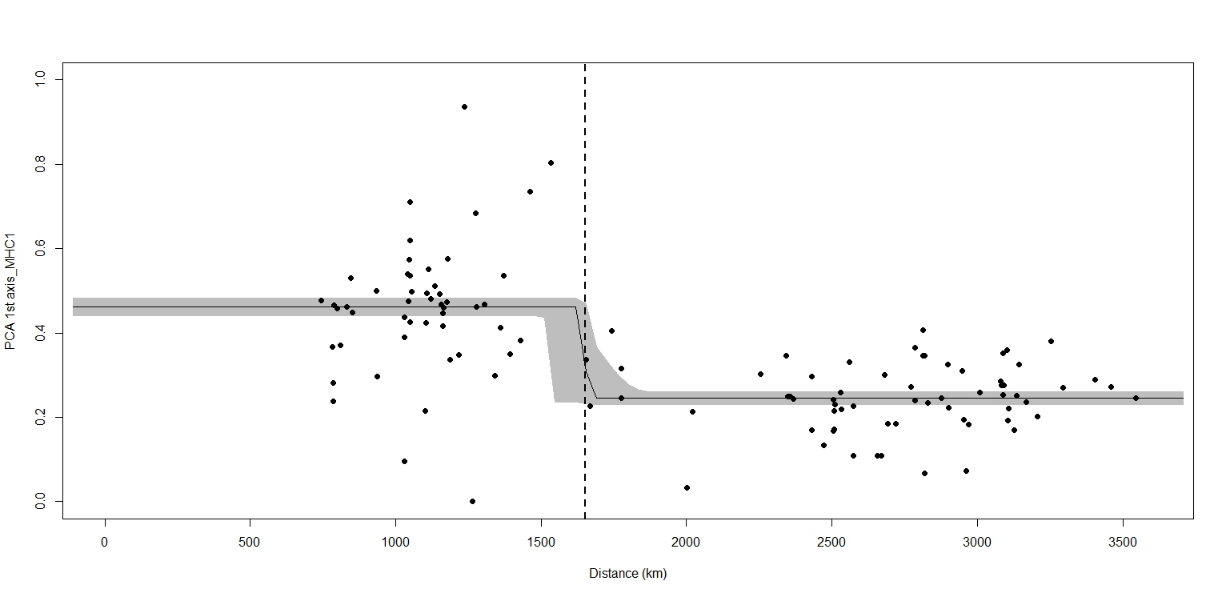 | 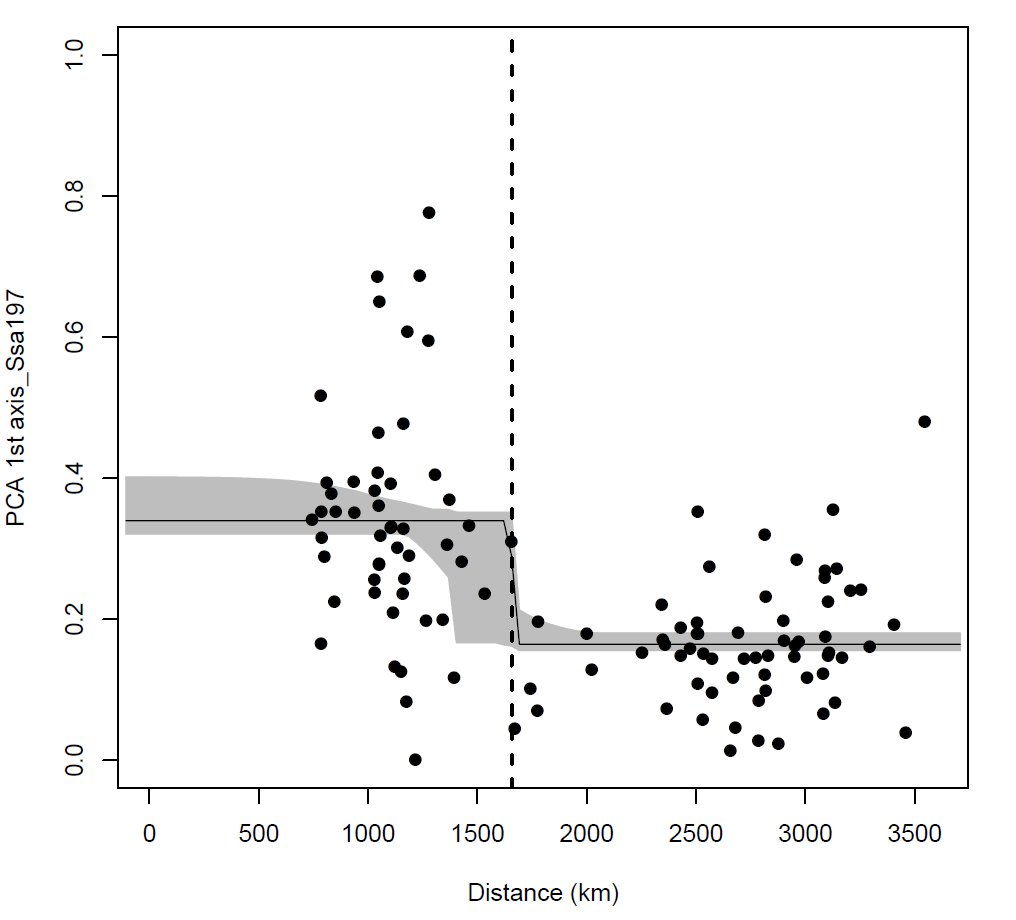 |
| 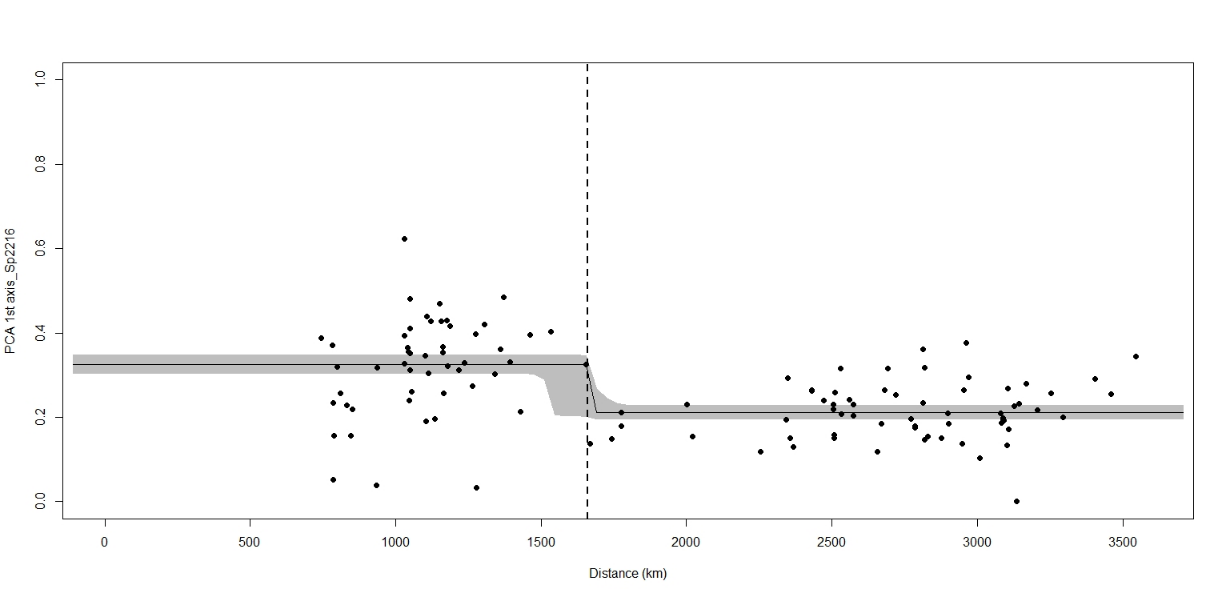 | 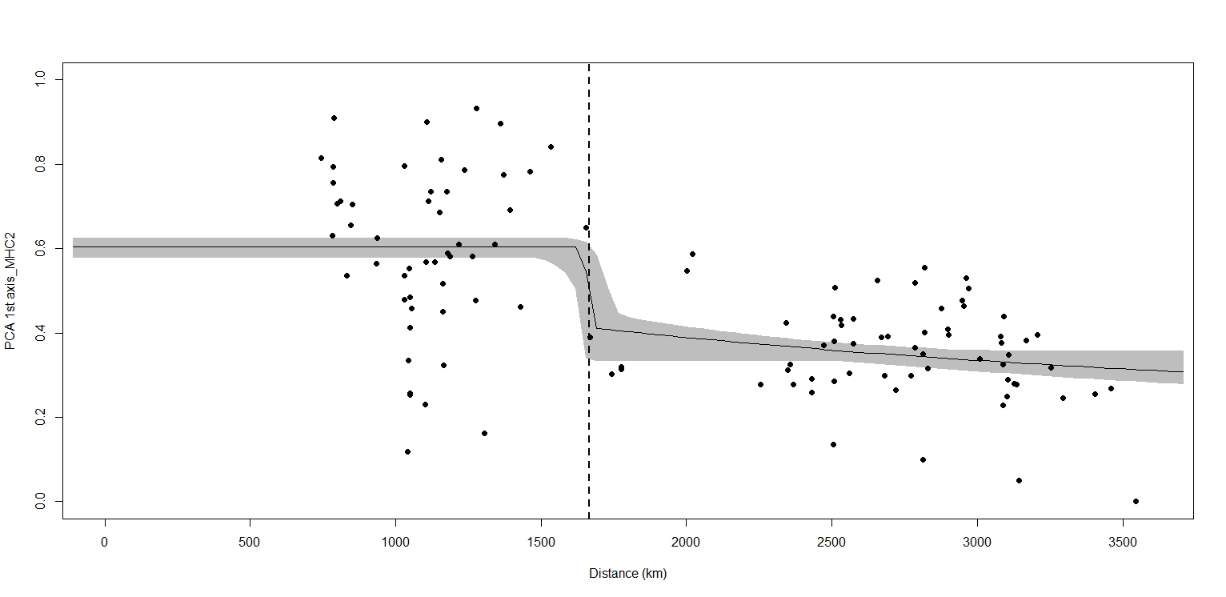 |
| 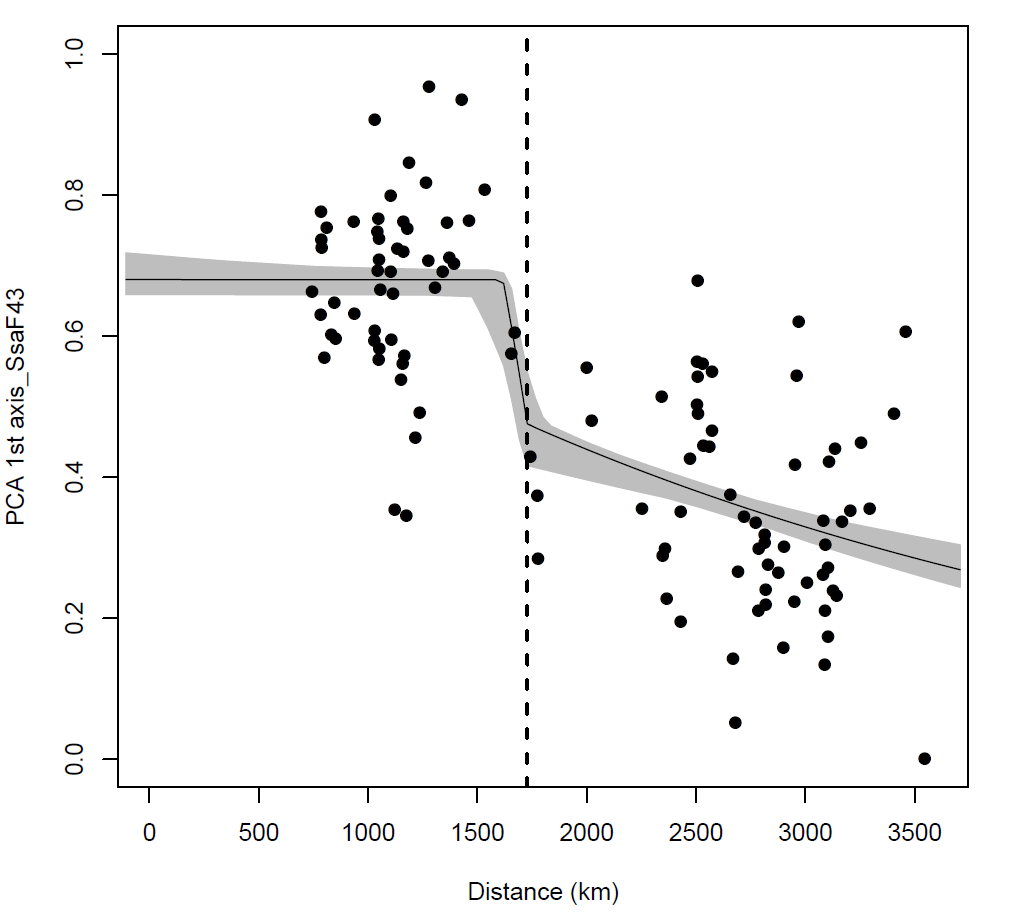 | 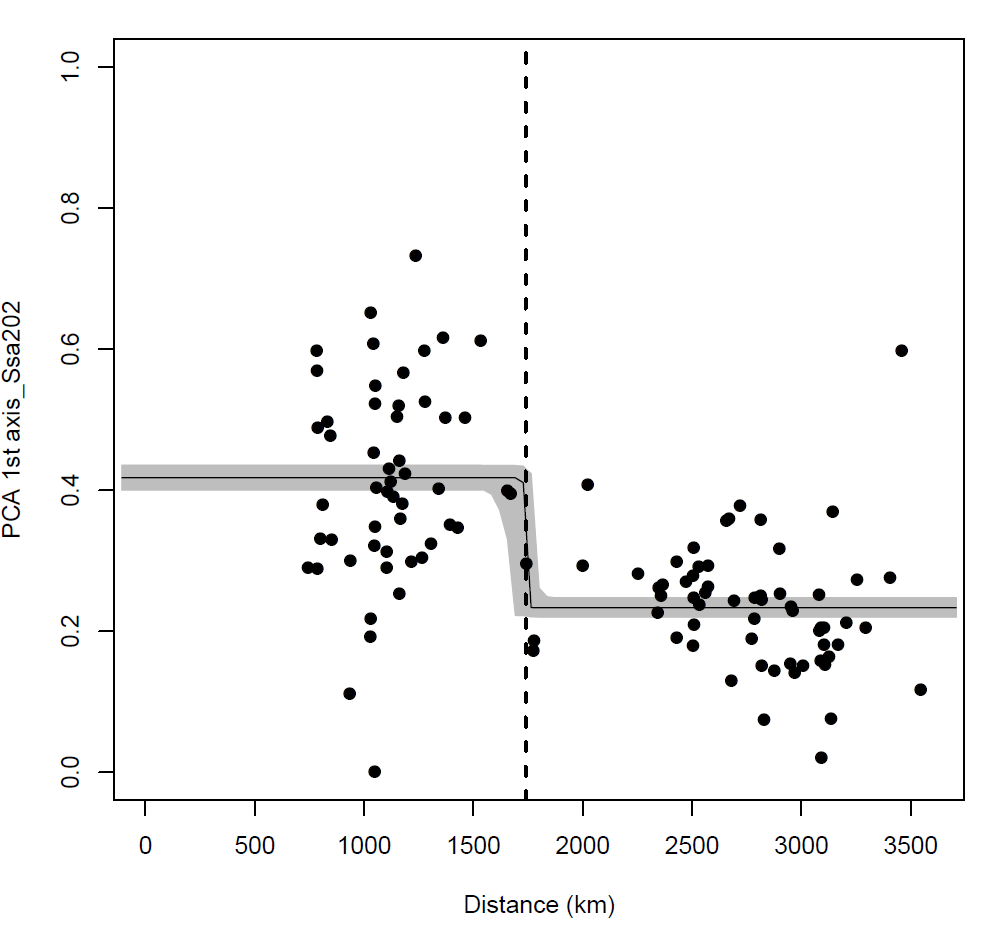 |
| 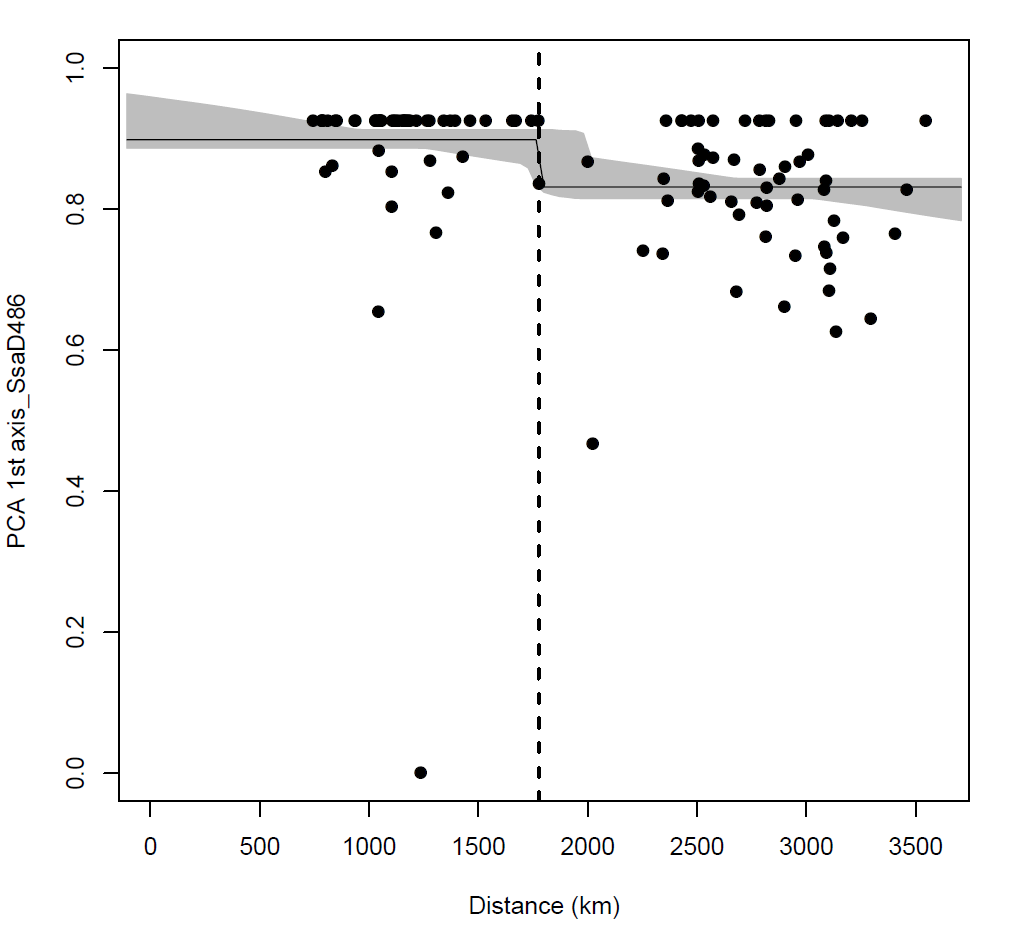 | 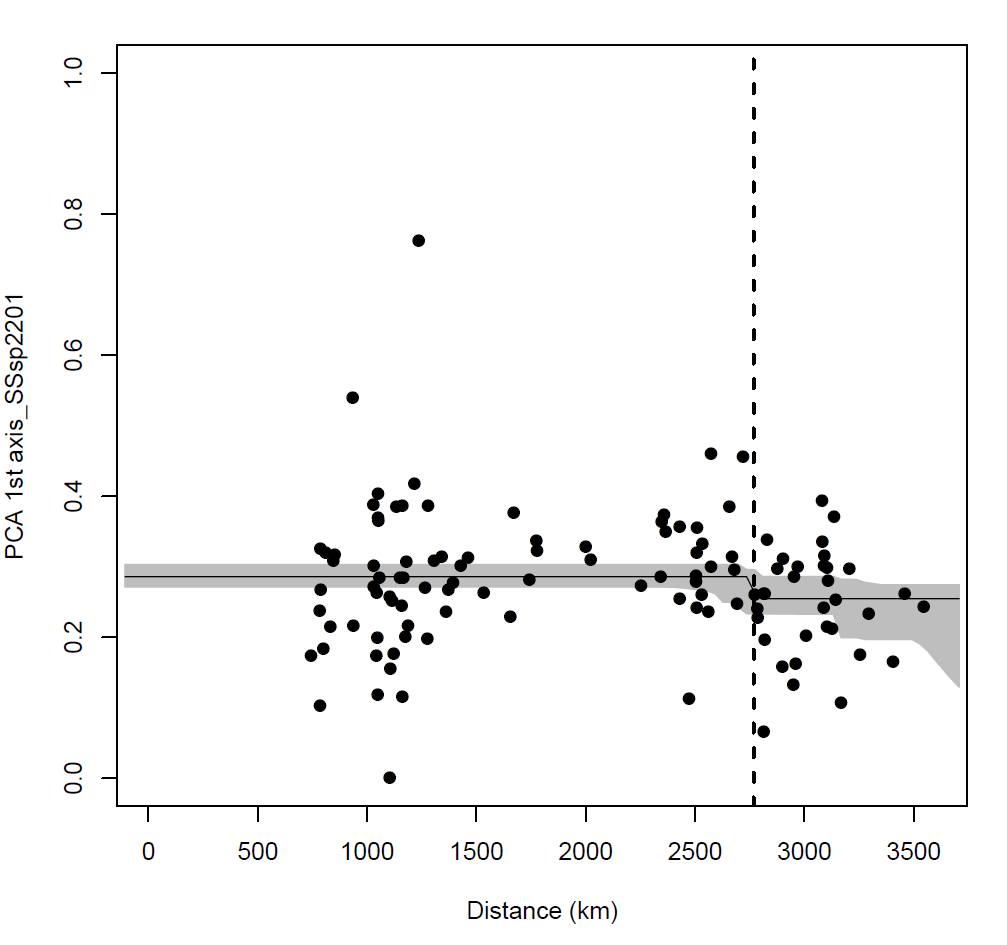 |

Fig. S1. Graphic representation of the clines for each of the markers along a geographic transect of circa 3600 km starting in the River Unya. Clines are shown with a grey shadow depicting the fuzzy 95% credible cline region as returned by the function hzar.plot.fzCline in HZAR, and centre of each cline is represented by a vertical dashed line.
